# SON-1010: an albumin-binding IL-12 fusion protein that improves cytokine half-life, targets tumors, and enhances therapeutic efficacy

**DOI:** 10.3389/fimmu.2024.1493257

**Published:** 2024-12-04

**Authors:** John K. Cini, Richard T. Kenney, Susan Dexter, Stephen J. McAndrew, Rukiye-Nazan Eraslan, Rich Brody, Darrel J. Rezac, Rebecca Boohaker, Suzanne E. Lapi, Pankaj Mohan

**Affiliations:** ^1^ Sonnet BioTherapeutics, Inc., Princeton, NJ, United States; ^2^ Disease Modeling and Oncology, Invivotek, Hamilton, NJ, United States; ^3^ InfinixBio, Inc., Athens, OH, United States; ^4^ Latham Biopharm Group, Inc., Elkridge, MD, United States; ^5^ Southern Research, Birmingham, AL, United States; ^6^ Radiology, Chemistry, and Biomedical Engineering, University of Alabama, Birmingham, AL, United States

**Keywords:** interleukin-12, tumor microenvironment, FcRn, SPARC, immunomodulation, fully human albumin binding (FHAB) domain, interferon gamma, immunotherapy

## Abstract

**Background:**

Cytokines have been promising cancer immunotherapeutics for decades, yet only two are licensed to date. Interleukin-12 (IL-12) is a potent regulator of cell-mediated immunity that activates NK cells and interferon-γ (IFNγ) production. It plays a central role in multiple pathways that can enhance cancer cell death and modify the tumor microenvironment (TME). Attempts to dose rIL-12 were initially successful but IFNγ toxicity in Phase 2 complicated further development in the late 1990s. Since then, better dosing strategies have been developed, but none have achieved the level of cancer control seen in preclinical models. We set out to develop a novel strategy to deliver fully functional IL-12 and other biologics to the TME by binding albumin, taking advantage of its ability to be concentrated and retained in the tumor.

**Methods:**

Single-chain variable fragments (scFv) were identified from a human phage display library that bound human, mouse, and cynomolgus macaque serum albumin, both at physiologic and acidic conditions. These were taken through a series of steps to identify strongly binding molecules that don’t interfere with the normal physiology of albumin to bind FcRn, giving it prolonged half-life in serum, along with SPARC/GP60, which allows albumin to target the TME. A final molecule was chosen and a single mutation was made that minimizes the potential for immunogenicity. This fully human albumin-binding (F_H_AB^®^) domain was characterized and manufacturing processes were developed to bring the first drug candidate into the clinic.

**Results:**

Once identified, the murine form of mIL12-F_H_AB was studied preclinically to understand its mechanism of action and biodistribution. It was found to be much more efficient at blocking tumor growth compared to murine IL-12, while stimulating significant IFNγ production with minimal toxicity. SON-1010, which uses the human IL-12 sequence, passed through all of the characterization and required toxicology and is currently being studied in the clinic.

**Conclusions:**

We identified and developed a platform technology with prolonged half-life that can target IL-12 and other immune modulators to the TME. Safety and efficacy are being studied using SON-1010 as monotherapy and in combination with checkpoint blockade strategies.

## 1 Introduction

Interleukin-12 (IL-12), first discovered in the late 1980s, is a multifunctional heterodimeric cytokine naturally produced by activated phagocytes and dendritic cells in response to bacterial or parasitic antigenic stimulation (1, 2). IL-12 is a key regulator of cell-mediated immunity that is expressed *in vivo* as p40 and p35 subunits joined by a disulfide bond that migrates as a 70 kDa molecule in its native state. However, recombinant human IL-12 is typically produced as a single polypeptide chain with the subunits joined by a Gly_6_Ser linker (3). IL-12 has multiple effector functions that bridge innate and adaptive immune responses in cancer (4) to a) induce the differentiation of naïve CD4^+^ T cells to become Th1 cells (5), b) increase the activation and cytotoxic capacities of CD8^+^ T and NK cells by promoting the expression of cytotoxic mediators and cytokines, especially interferon gamma (IFNγ), c) inhibit the differentiation of Treg cells, and d) inhibit or reprogram immunosuppressive cells, such as tumor-associated macrophages (TAMs) and myeloid-derived suppressor cells (MDSCs) (6). Directly or with the help of IFNγ, IL-12 also activates the immune function of B cells with increased production of IgG2a (7).

Control of tumor growth by IL-12 in mouse models is primarily achieved by activation of the effector Th1 response, which is required for activation of cytotoxic T and NK cells and tumor clearance (8). This Th1 response can be assessed by measuring the induction of significant amounts of systemic IFNγ, which itself is cytostatic/cytotoxic and antiangiogenic. IL-12 stimulates the expression of two IL-12 receptors, IL-12Rβ1 and IL-12Rβ2, which act to maintain the expression of STAT4, a critical intracellular protein involved in IL-12 signaling in NK cells. The enhanced functional response is demonstrated by IFNγ production and killing of target cells. Tumor necrosis factor-alpha (TNFα) from T cells and NK cells is also stimulated to reduce IL-4-mediated suppression of IFNγ (9) and upregulate tumor cell MHC I and II expression for enhanced recognition and lysis (10, 11). In addition, IL-12 transforms pro-tumor M2 MDSCs into inflammatory M1 antigen presenting cells (APCs), leading to recovery of their macrophage function (12). The overall sequence of effects demonstrates the potential to turn “cold” tumors into “hot” tumors (6, 13). The effectiveness of immune modulators depends on the interplay between the physical properties of the drug and the tumor microenvironment (TME), including serum pharmacokinetic (PK) properties, vessel permeability, immune cell infiltration, and tumor retention time (14–16).

IL-12 and related compounds have been extensively studied in cancer and immunotherapy indications (8, 17). However, recombinant interleukins have had limited clinical success in cancer owing to their short circulating half-life, inefficient TME targeting, and requirement for frequent dosing, often leading to substantial systemic toxicities. Delivery of drugs or nanocarriers to the tumor has been one of the most challenging aspects. For instance, a recently published study noted only 0.0014% of the targeted nanocarriers accumulated in the TME (18). Several approaches have been developed that use albumin as a carrier to extend half-life and target tumors (19–21). Incorporating an albumin strategy to help target and retain IL-12 in the tumor tissue has the potential to be more effective than injecting recombinant IL-12 alone. Furthermore, this strategy could also decrease the risk of toxicity, resulting in a broader therapeutic index.

**Figure 1:**
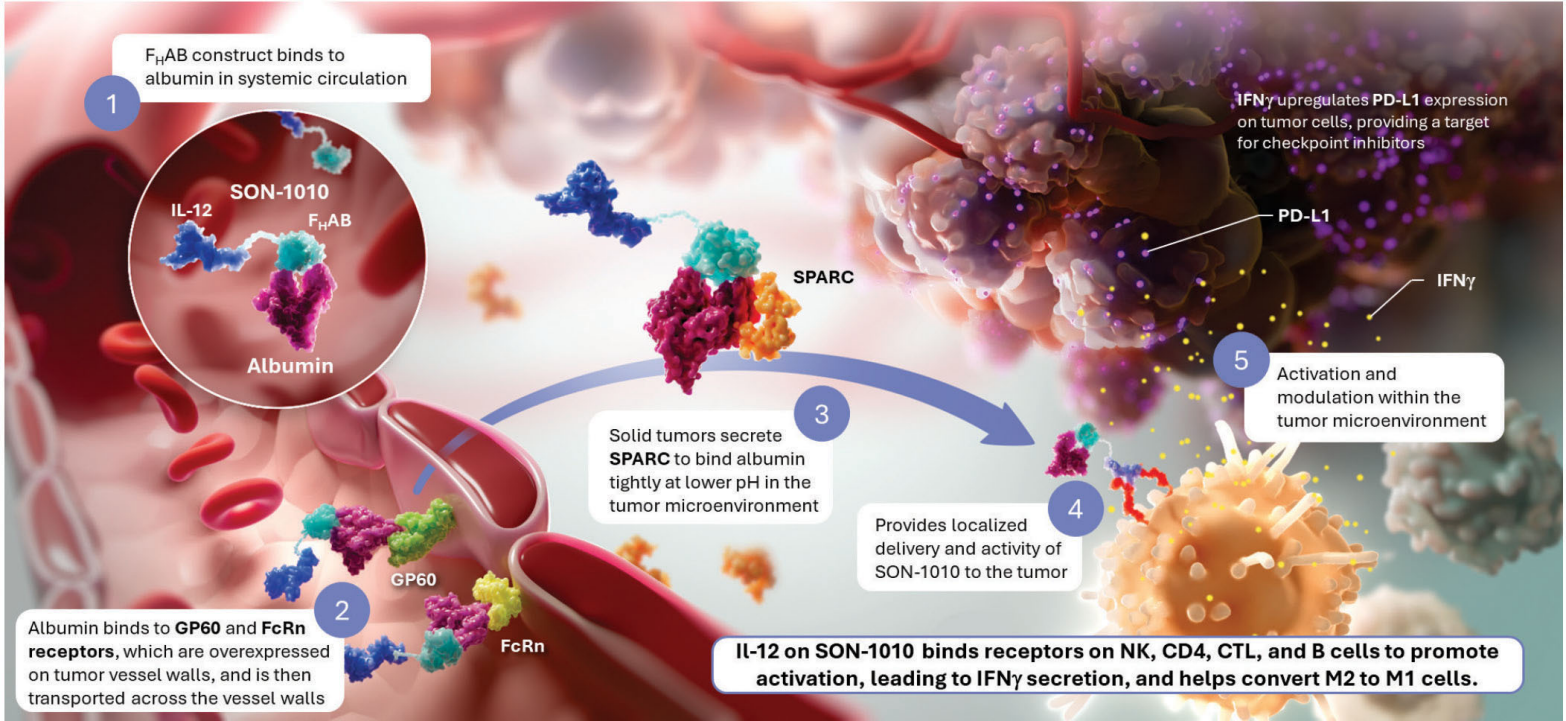
SON-1010 Binds Albumin to Transport IL-12 to the Tumor Microenvironment SON-1010 includes single-chain hIL-12 as a payload that is genetically linked to the V_H_ domain on the F_H_AB. **(1)** The scFv domain binds to albumin at both physiological and acidic pH, in a region that doesn’t interfere with its binding to FcRn, GP60, or SPARC. Albumin binds systemically to FcRn, which extends its half-life, and SON-1010 shares albumin’s PK by hitchhiking in the circulation. **(2)** The entire complex can be carried into the tumor tissue through the bloodstream, where FcRn and GP60 receptors are upregulated, to be transported across the endothelium into the acidic TME. **(3)** Once there, albumin binds tightly in dynamic equilibrium via its interaction with SPARC (19), which is overexpressed in the TME. **(4)** Retention of SON-1010 in the TME provides localized delivery of bioactive IL-12 to promote activation. **(5)** The rIL-12 cytokine domain can activate resident immune cells and recruit more cells, upregulating the expression of IFNγ from NK, CD4^+^, and CD8^+^ T cells, which then upregulates PD-L1 expression on tumor cells and antibody production from B cells. IL-12 also helps switch M2 macrophages to the M1 phenotype (12). FcRn, neonatal Fc receptor; GP60, albondin/glycoprotein 60; F_H_AB, fully-human albumin binding; PD-L1, programmed cell death ligand 1; PK, pharmacokinetics; SPARC, secreted protein acidic and rich in cysteine.

To address these challenges, we developed and validated a platform technology that prolongs cytokine half-life and targets the TME by linking rIL-12 to a single-chain variable fragment (scFv) that is a fully human albumin-binding (F_H_AB^®^) domain (22), resulting in a fusion protein designated as SON-1010 (**Figure 1**). Albumin has a serum concentration of 42-54 g/L in humans and an extremely long half-life of 19-21 days, which is credited to its reduced filtration in the kidney and its recycling via binding to the neonatal Fc receptor (FcRn) at low pH (23). The F_H_AB domain is carried by albumin in the serum and exploits its physiological recycling and accumulation in tumors, as albumin also binds efficiently to proteins such as FcRn, GP60, and SPARC, which are overexpressed in many solid tumor microvessels (19, 24). SON-1010 binding provides for the concentration and retention of the drug molecule as a complex that is bound to albumin both in serum and at lower pH in the TME, via enhanced penetration and retention (EPR) (25). The rIL-12 p40 and p35 subunits are linked with Gly_6_Ser, while the V_H_ and V_L_ scFv domain is linked using (Gly_4_Ser)_3_. IL-12 is linked to the F_H_AB domain with (Gly_4_Ser)_5,_ which is a commonly used polypeptide chain that has been shown to be water-soluble, non-immunogenic, and resistant to a multitude of proteases (26). The 5x repeat length was shown to be optimal for flexibility, resulting in limited steric hindrance and reduced aggregation. SON-1010 retains the full biological activity of IL-12 in this configuration.

SON-1010 was designed to (a) increase the half-life of the F_H_AB complex; (b) increase selective penetration in the tumors versus other tissues; and (c) maintain the pharmacologic activity of the IL-12 therapeutic payload attached to the F_H_AB via flexible linkers. The objective was to realize improved immunological potency of IL-12 by overcoming the challenges associated with the native molecule, collectively leading to fewer systemic toxicities and better clinical effectiveness. The anti-albumin-linked construct provides a means for the rIL12 protein to maintain a tumor-specific, efficacious, therapeutic concentration over an extended period, while decreasing potential toxicity and contributing to the drug’s safety.

The initial clinical focus is on immunotherapy of solid tumors; however, the technology is well-suited for drug development across the spectrum of human diseases, as a number of different biologic payloads can be added to the scFv. Currently, SON-1010 (IL12-F_H_AB) is being studied in patients with advanced solid tumors and platinum-resistant ovarian cancer (PROC) using a multiple ascending dose (MAD) design in the first-in-human study SB101 (NCT05352750) (27), in healthy volunteers using a single ascending dose (SAD) design in study SB102 (NCT05408572) (28), and in patients with advanced solid tumors and PROC in combination with atezolizumab in a dose-escalation format, followed by a randomized comparison with monotherapy or the standard-of-care in study SB221 (NCT05756907) (29). The sections that follow describe the F_H_AB mechanism of action, as well as the PK and pharmacodynamic (PD) studies demonstrating these improvements over IL-12 alone and the studies used to support clinical development.

## 2 Materials and methods

### 2.1 Selection of an anti-HSA scFv

**Figure 2:**
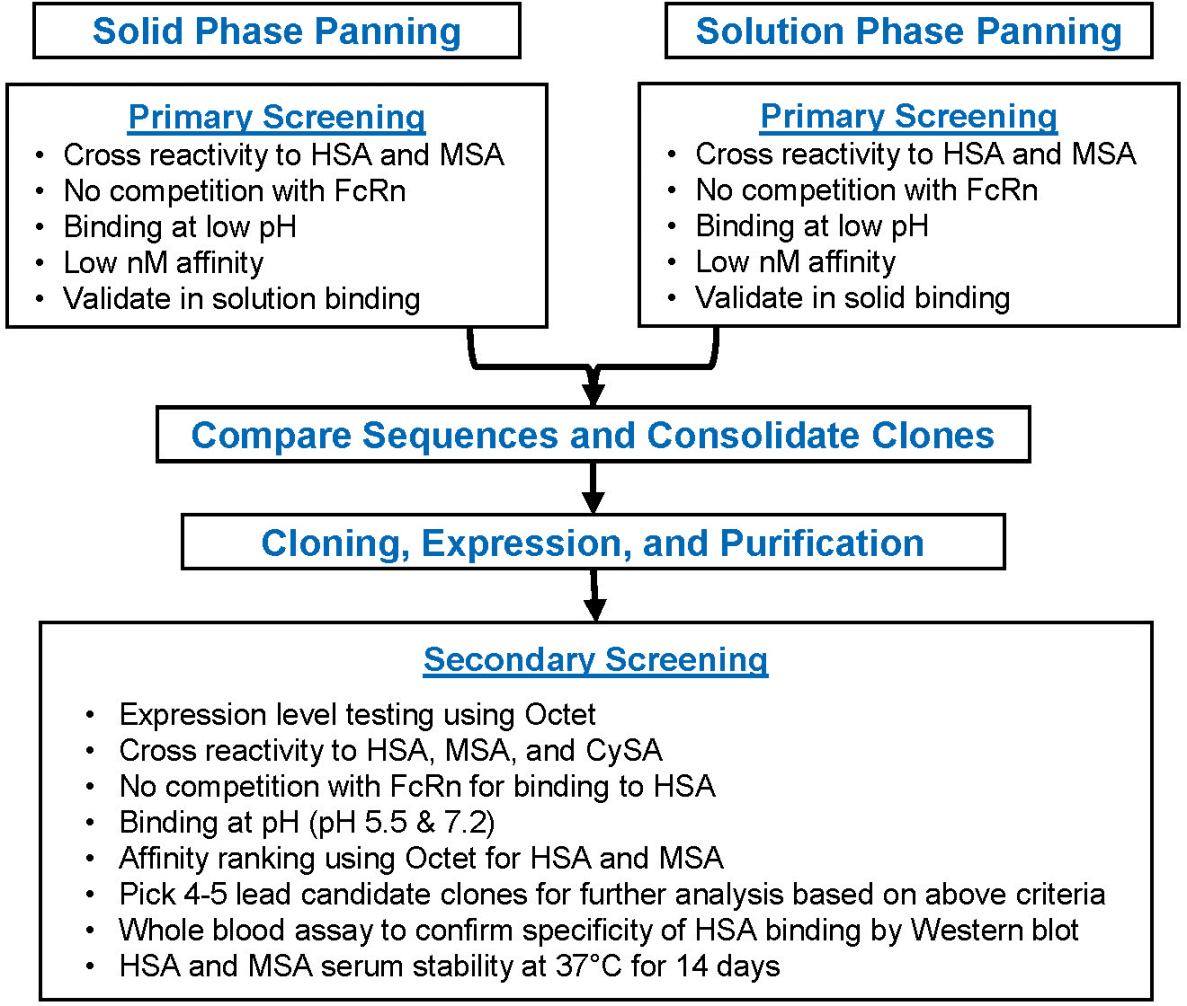
Candidate Selection Flowchart for the F_H_AB domain After conducting solid and solution phase primary screening for scFv clones, a series of bioactivity selection criteria were established to ensure binding to albumin at lower pH and an increase in the half-life. HSA, human serum albumin; M, mouse serum albumin; CySA, cynomolgus serum albumin.

A human scFv phage display library (Xoma, Emeryville, CA, Cat# XOMA040) was used to identify scFv’s that bind human serum albumin (HSA) (22). The library is a naïve (non-immunized) phage display library of human antibody V_H_ and V_L_ sequences cloned from 33 healthy donors. Sequence diversity over 10^12^ in the library raises the probability of obtaining high affinity binders from the library. To ensure selection of a wide array of HSA-specific scFv’s, both solid phase and solution phase panning methods were used (**Figure 2**).

Albumin becomes highly modified in blood and specific properties of serum albumin purified from human blood can vary from lot to lot, so recombinant HSA (rHSA) was used as the panning antigen. The library was initially screened using solid-phase panning by adding aliquots to plastic wells containing immobilized target rHSA, followed by extensive washing with RPMI-1640 (ATCC Cat# 30-2001) to remove non-specifically bound phage. Three rounds of solid phase panning against 150 nM, 75 nM, and 30 nM of rHSA, respectively, were performed with the library and each subsequent round showed enrichment for rHSA binding. ScFv periplasmic extracts (PPE) of 3^rd^ round binders were tested in an ELISA against rHSA and analyzed using Xabtracker software (Xoma). The threshold set for positive binders was a signal 3-fold higher than the negative PPE control. Given that the final HSA binder should also bind serum HSA (sHSA), the positive rHSA binders were pooled and analyzed for their binding to sHSA at a K_d_ of ~20-60 nM. In addition, to support mouse proof of concept studies the selected candidates were screened for cross reactivity to mouse serum albumin (MSA) for binding at a K_d_ of ~10-30 nM. Candidates were then selected for meeting the following criteria: a) the variable heavy chains belonged to a V_H_3, V_H_4 or V_H_5 family (the V_H_3 is the preferred V_H_ family, as it has been shown to exhibit very high stability in the scFv format) (30); b) the signal to negative control ratio for HSA ELISA binding was over 20-fold; c) the signal to negative control ratios for MSA in ELISA was over 20-fold; and d) they bind with high affinity to the human FcRn at pH 5.5 (representing the acidic TME) and at physiologic pH 7.2.

A three-round solution-based panning method was also used to select scFv’s that bind to HSA in its most native form, utilizing direct-capture magnetic bead separation to harvest high affinity HSA-specific scFv phage binders. Prior to panning, the XOMA040 scFv library was deselected against streptavidin coated magnetic M270 Dynabeads (ThermoFisher Scientific, Cat# 65306) to deplete any streptavidin-specific binders from the antibody pool. Simultaneously, 200 pM of biotinylated rHSA were coupled to 1.2 x 10^8^ streptavidin beads (200 ul) via non-covalent binding interaction between biotin and streptavidin (K_d_ 10^-4^ nM). The deselected phage library was incubated with HSA-coupled magnetic beads for 1 hour at RT. The phage-HSA-bead complexes were captured and separated from non-complexed phage via magnet separation. Phage-HSA-bead complexes were washed extensively with RPMI to remove non-specific binders. The HSA-specific phage were eluted from the antigen-bead complex with triethylamine (TEA). Eluted selected phage were neutralized in Tris-HCl and used to infect TG-1 bacteria for subsequent rounds of panning against 50 pM and 200 pM of biotin-HSA, respectively.

To extend the half-life of the anti-albumin scFv’s and to take advantage of the tumor accumulating properties of albumin, demonstration of binding by the scFv’s to HSA at low pH, as well as their lack of binding to the FcRn binding domain on HSA, was required. A competition ELISA was used to assess binding to FcRn, which binds to HSA in a pH-dependent manner, as scFv and FcRn binding to HSA can be detected simultaneously. Detection of dual signals indicate binding to HSA and the lack of overlapping scFv and FcRn binding domains on HSA, which allows scFv’s that are bound to albumin to undergo the physiologic FcRn-mediated endosomal recycling. In the competition assay, ELISA plates were coated with HSA and incubated with 2 nM FcRn for 1h, then for another hour after adding each biotinylated scFv-PPE detection antibody (ThermoFisher Cat#CUST77216). After washing, samples were incubated with Streptavidin-HRP (ThermoFisher 21130), washed four times, and visualized with TMB (Sera Care Cat#5120-0083) for 10 minutes before quenching with 0.05 mL 1 M HCl and reading the plate at 450 nm. The K_d_ value was determined at each concentration of added albumin. No binding of rFcRn was observed at pH 7.4 (31) and weak binding occurred at pH 6. However, the strongest binding was demonstrated at pH 5.5, which was thereby selected as the lower pH for the assay.

In total, 15 unique clones were selected from solution and solid phase panning that did not compete with FcRn binding to albumin. Clones were confirmed for their binding to HSA in solution on Octet^®^ (Sartorius, Goettingen, Germany), then were sequenced and subsequently assayed for target concentration-dependent binding, pH stability, FcRn binding interference, and kinetic binding. In particular, candidate scFv’s were chosen for their ability to bind HSA (K_d_ ~20-60 nM), MSA (K_d_ ~10-30 nM) and cynomolgus serum albumin (CySA) (K_d_ ~20-60 nM), both at low pH (pH 5.5) and neutral pH (pH 7.2). Several albumin binding clones were selected based on these criteria; of these, Al0 was the only clone identified by both panning techniques and it was finally selected based on higher expression levels and best activity profile. After further testing, clone Al0 was mutated to eliminate regions that could putatively cause immunogenicity by iTOPE™ and TCED™ analysis of putative CD4^+^ T-cell epitopes (32, 33). From these Al0 variants, Al0m3 was selected as the lead based on its high affinity for serum albumin (**Supplementary Figure 1**).

Phage sequences were initially cloned into a pET-22b vector (Novagen Cat# 69744) that contains a 6-His tag sequence at the C-terminus, preceded by a human or mouse single-chain IL-12 sequence. The 6-His tag was utilized for affinity purification, where an immobilized metal affinity chromatography (IMAC) column was used to separate the His-tagged scFv protein from other harvest media components. Following IMAC purification, the eluted scFv solution was further purified using a preparative size exclusion column (SEC) that separates the scFv construct from protein aggregates, fragments, and non-scFv proteins that non-specifically bound to the IMAC column and co-purified with the target protein.

Each of the final fusion protein nucleotide sequences were subsequently inserted into pSecTag2A vector (Life Tech Cat# V900-20), after the vector was modified to remove all of the original tags and extra restriction enzyme sites, with mouse or human IL-12 sequence inserted 5’ to the scFv sequence. Human IL-12 sequence uses a G6S subunit linker between the two subunits, while the mouse version uses a (G4S)_3_ linker (**Supplementary Figure 2**). Proteins were produced in either HEK293T or Chinese Hamster Ovary (CHO) cells. The purification process was revised for this new vector (which lacks the 6-His tag) to start with a heparin column, as IL-12 has an affinity to heparin. The IL12-F_H_AB molecule was further purified using mixed mode size-exclusion chromatography on a ceramic hydroxyapatite column, which provides adequate removal of scFv aggregates and process impurities.

### 2.2 Generation of IL12-F_H_AB (SON-1010) batch lots

The IL12-F_H_AB proprietary manufacturing process consists of upstream cell culture by fed-batch or continuous perfusion (at Abzena, San Diego, CA or Enzene, Pune, India, respectively), followed by downstream purification. A vial of the master cell bank is thawed and the cells are transferred into culture medium. A series of expansion phases are then performed by culturing cells at increasing volume in shake flasks and cell bags. Once the target cell density is achieved, the culture is used to inoculate the production bioreactor, which was operated in a fed-batch mode (Abzena, San Diego, CA) to make mIL12-F_H_AB for preclinical studies or hIL12-F_H_AB (also called SON-1010) for the initial clinical studies, and in a continuous perfusion mode (Enzene Biosciences Ltd., Chakan Pune, India) to make hIL12-F_H_AB for later stage clinical use. In the latter, alternating tangential-flow filtration is used for cell retention and the introduction of fresh media.

When harvest criteria are met, the culture is processed through a depth filter train to remove cells and cell debris. SON-1010 is captured using affinity chromatography with on-column viral inactivation. A viral filtration step is followed by ultrafiltration/diafiltration concentration and formulation into final drug substance (DS), then diluted to the final concentration and stored as a frozen liquid or lyophilized drug product (DP). While downstream processing was initially done as unit operations, the approach was changed with further development to a continuous process from bioreactor through capture and polishing steps to the final viral filtration step and final formulation.

### 2.3 *In vitro* characterization of the F_H_AB

#### 2.3.1 Testing of production lots

Native IL-12 has a predicted molecular weight of 57 kDa based on the amino acid sequence. Due to glycosylation, an apparent molecular mass of 65-70 kDa is observed on SDS-PAGE gel under non-reducing conditions. The predicted mass of the F_H_AB is ~ 26 kDa and that of IL12-F_H_AB without glycosylation is ~ 85 kDa. Glycosylation causes an apparent increase in molecular mass, causing IL12-F_H_AB to appear as 75-100 kDa under non-reducing conditions. Comparison of the DS made under Good Manufacturing Practice (GMP) conditions using the fed-batch process versus the continuous perfusion process was performed with standard assay techniques that included non-reduced peptide mapping of the column elution patterns, peptide mapping of the disulfide linkages, and far-UV CD spectroscopy for secondary structure analysis, along with fluorescence and near-UV CD spectroscopy for tertiary structure analysis. SDS-PAGE analysis was performed using 4-20% Bis-Tris pre-cast gel for samples under reducing and non-reducing conditions. Size exclusion chromatography was performed to check for the presence of size variants using a TSK gel column with detection at 214 nm. Reverse phase chromatography was used to check for hydrophobic variants using a Pursuit-3-diphenyl column with detection at 214 nm.

HSA binding was confirmed by ELISA analysis. IL12-F_H_AB was captured on plates coated with HSA and then was detected using an anti-IL-12 antibody. IL-12 receptor binding activity and signal activity was determined using a HEK-Blue™ IL-12 cell-based assay (34). These cells have the human genes for the IL-12 receptor and the genes of the IL-12 signaling pathway inserted into the human embryonic kidney HEK293T cell line. Furthermore, these cells express a STAT4-inducible SEAP reporter gene. As a result, binding of IL-12 to the IL-12 receptor on the surface of these cells triggers a signaling cascade, leading to the activation of STAT-4 with the subsequent production of SEAP, which is secreted in the supernatant and detected quantitatively with QUANTI-Blue™ substrate. The intensity of the color produced is then read at 625-655 nm and is directly proportional to the original quantity of the IL12-F_H_AB.

#### 2.3.2 Further characterization of binding

The F_H_AB technology was designed to promote targeting of the TME after binding to albumin *in vivo*, which is particularly helpful when intratumoral levels of SPARC are elevated. At a normal serum pH of 7.2, binding of SPARC to human albumin is relatively weak, with a K_d_ of ~35 µM using Surface Plasmon Resonance (SPR) analysis with histidine-tagged SPARC immobilized on a solid support (35). Binding constants were determined to show F_H_AB binding in various conditions at both physiologic, as well as at an acidic pH to emulate the TME (34).

### 2.4 *In vivo* characterization of the F_H_AB

#### 2.4.1 Tumor-targeting by F_H_AB in the 4T1 mouse mammary tumor model

An initial proof-of-concept (POC) study was designed to show that the F_H_AB scFv, when bound to albumin *in vivo*, increases retention time in the tumor. For this study, a 4T1 mouse mammary tumor model that overexpresses TGFß was used to demonstrate F_H_AB binding to tumor-associated albumin and SPARC when an anti-TGFß scFv (K_d_ with TGFß at ~ 10nM) was used alone or was linked to the F_H_AB. A low dose (1 x 10^4^ cells) of 4T1 breast cancer cells were injected into the BALB/c mammary fat pad in 36 mice. Once tumors reached ~150 mm^3^, Western blot analysis (detecting the HIS-tag) was conducted on tumor extracts from 3 mice/group that had been terminated at 0.5, 4, 12, or 24 hours after IV injection of 100 µg/mouse of the F_H_AB, anti-TGFβ (selected from the same human scFv phage display library), or anti-TGFβ-F_H_AB.

#### 2.4.2 Assessment of mIL12-F_H_AB in the B16-F10 melanoma model

Pharmacology studies were performed using single-chain mouse IL-12 as the F_H_AB-linked payload in the B16-F10 mouse model to evaluate accumulation, assess antitumor activity, compare the safety and pharmacologic activity, and to determine retention time on target. The B16-F10 model is considered to be an immunologically “cold” tumor model that aggressively grows tumors and does not respond well to checkpoint inhibitors. In these studies, B16-F10 cells were cultured in Dulbecco’s Modified Eagle Medium (DMEM) with 10% fetal bovine serum (FBS) and 0.1% gentamicin. For the tumor model, B16-F10 cells (5 x 10^5^/mouse in 0.2 mL of serum-free media DMEM) were implanted to C57BL/6 female mice via mammary fat pad adjacent the inguinal lymph node for each experiment. Once the tumors reached ~150 mm^3^ about a week later, treatments were administered as a single IV dose in the tail vein, establishing Day 0 for every study. To investigate the comparative efficacy of tumor suppression by mIL-12 or mIL12-F_H_AB in this model, groups of melanoma-bearing C57BL/6 mice were administered different dose levels of mIL-12, mIL12-F_H_AB, or buffer as a placebo. Doses were compared based on a molar ratio of 1.5 (70 kDa for mIL12 versus 105 kDa for mIL12-F_H_AB), hence 10 µg mIL-12 is the molar equivalent of 15 µg mIL12-F_H_AB.

The serum half-life of mIL12-F_H_AB had been shown to be increased nearly 4-fold in C57BL/6 mice at 24 hours compared to mIL-12 using a HIS-tag ELISA (34). Its accumulation in spleen and tumor was studied using the same ELISA at 24 hours in the B16-F10 mouse melanoma model. In other studies, tumor growth measurements (mm^3^) and body weight (g) were measured intermittently for the duration of each study. Tumor growth alone was measured as a surrogate for the decision to end the study (when 50% of the last group had tumors that were >2,000 mm^3^).

### 2.5 Biodistribution

#### 2.5.1 ^89^Zr-labeling of mIL-12 and mIL12-F_H_AB

A radiolabeling study was designed to examine the biodistribution of mIL-12 and mIL12-F_H_AB in both a non-tumor-bearing and a B16-F10 tumor-bearing mouse model, assuming the mIL12-F_H_AB might target tumors more effectively (36, 37). The protein products were expressed in the CHO mammalian expression system and purified to > 95% homogeneity by SDS and HPLC. Characterization of the mIL-12 and mIL12-F_H_AB molecules for this study showed activity by the IL-12-specific HEK-blue assay using STAT4 as reporter gene. The F_H_AB domain was shown to bind human or mouse albumin in concentrations ranging from 30-50 nM. The objectives of the study were to: 1) radiolabel the mIL12-FHAB with limited disruption of the albumin binding functionality; 2) determine the longitudinal distribution and clearance of mIL12-F_H_AB relative to mIL-12 alone; and 3) determine the tumor accumulation and retention of mIL-12 and mIL12-F_H_AB over time. Each molecule was covalently modified first with the bifunctional chelator, p-SCN-deferoxamine (DFO), then radiolabeled with ^89^Zr, which has a half-life of 78.4 hours.

The active agents were conjugated with DFO using 10μg (0.2 mg/mL in PBS) of IL-12 or IL12-F_H_AB mixed with DFO-NCS (2.5 μg/μL in DMSO). Then 10 μL of 1 M Na_2_CO_3_ was added to mixture and incubated for 1h at 37°C. DFO conjugated IL-12 or IL12-F_H_AB was loaded onto a 7K MWCO Zeba desalting spin column to remove the excess unbound DFO. ^89^Zr-oxalate was produced at the University of Alabama at Birmingham and neutralized with 2 M NaOH/HCl (38). 10μg (0.2 mg/mL in PBS) of DFO-IL-12 or DFO-IL12-F_H_AB was mixed with 0.1 mCi of neutralized ^89^Zr-oxalate. The mixtures were incubated for 1h at 37°C. Radiochemical purity was checked with iTLC using 50mM DTPA.

Titration of the ^89^Zr allowed for minimal labeling to acquire an imaging signal, with the conjugation of DFO likely occurring on a primary amine on the IL-12 itself, rather than the F_H_AB-albumin interface. This was confirmed by a series of *in vitro* assessments of the DFO conjugate by ELISA and STAT4 phosphorylation status. Quantification of albumin binding via ELISA allowed for a comparison between the IL12-F_H_AB and IL12-F_H_AB-DFO conjugate. The presence of the F_H_AB domain resulted in a 60% recovery of IL12-F_H_AB and this was unchanged when the DFO was conjugated, indicating that the DFO linker was not affecting the albumin binding capacity. The complementary experiment, looking at binding capacity to anti-IL-12, demonstrated a concentration-dependent binding of the mIL12-F_H_AB to the immobilized antibody; however, the binding of the DFO to mIL-12 abrogated the signal. This effect may be due to the positioning and size of the DFO on mIL-12 acting as a steric inhibitor of binding. To ensure that the DFO was not affecting functionality, the phosphorylation status of STAT4 was assessed in naïve murine CD3^+^ PBMCs stimulated with mIL-12 alone, mIL12-F_H_AB, and mIL12-F_H_AB-DFO. The DFO did not significantly affect the ability for signal transduction through STAT4 phosphorylation. Indeed, the F_H_AB in both DFO and non-DFO conditions appears to retain better activity than mIL-12 alone.

#### 2.5.2 Experimental plan to assess biodistribution in mice

A total of 98 C57BL/6 female mice were split into 11 tumor-bearing and 3 control (tumor-naïve) groups (**Table 1**); an animal placement plan was used to ensure that mean tumor size was distributed equally across the tumored groups. The mice were initially implanted with 5 x 10^5^ B16-F10 cells/mouse in 0.1 mL of serum-free media DMEM via mammary fat pad adjacent the inguinal lymph node on Day 0. When the tumors reached ~ 125-250 mm^3^, the mice were assigned to 1 of the 11 tumor-bearing groups. Mice were then injected with 3.7 MBq (100 μCi) in 100 μL of test article via the tail vein.

**Table 1:** Tissue Biodistribution Experimental Plan.

Group	# of Animals	Test Article	Dose / Animal	Dose Volume	Dose Route	Imaging for Biodistribution	Euthanasia
Non-Tumored							
**1**	10	--	--	--	--	3, 12, 24, 48, 72h	72 hours
**2**	10	IL-12	10 ug	100µL	IV	3, 12, 24, 48, 72, 96h	96 hours
**3**	10	IL-12-F_H_AB	10 ug	100µL	IV	3, 12, 24, 48, 72h	72 hours
Tumor Efficacy							
**4**	5	IL-12	10 ug	100µL	IV	--	6 hours
**5**	5	IL-12-F_H_AB	10 ug	100µL	IV	--	6 hours
**6**	5	IL-12	10 ug	100µL	IV	--	24 hours
**7**	5	IL-12-F_H_AB	10 ug	100µL	IV	--	24 hours
**8**	5	IL-12	10 ug	100µL	IV	--	48 hours
**9**	5	IL-12-F_H_AB	10 ug	100µL	IV	--	48 hours
**10**	5	IL-12	10 ug	100µL	IV	96 hours	96 hours
**11**	5	IL-12-F_H_AB	10 ug	100µL	IV	96 hours	96 hours
Tumor Longitudinal							
**12**	4	--	--	--	--	--	Day 10
**13**	12	IL-12	10 ug	100µL	IV	--	Day 10
**14**	12	IL-12-F_H_AB	10 ug	100µL	IV	--	Day 10

Imaging studies were performed at the indicated times after dosing. PET and CT images were acquired on a Sofie GNEXT PET/CT scanner. The PET images (energy window 350-650 keV) were reconstructed using a 3D-OSEM (Ordered Subset Expectation Maximization) algorithm (24 subsets and 3 iterations), with random, attenuation, and decay correction. The CT images (voltage 80 kVp, current 150 μA, 720 projections) were reconstructed using a modified Feldkamp algorithm. Following image reconstruction, images were converted to standard uptake values (SUV) and regions of interest (ROIs) were drawn for select organs using the CT. All image processing was performed using VivoQuant (Invicro).

Biodistribution studies of mIL-12 and mIL-12-F_H_AB on control and tumor animal organs were performed as indicated. Following euthanasia, target organs (blood, heart, lung, spleen, liver, kidney, inguinal lymph nodes, tumor) were collected and assessed for radioactivity biodistribution. The radioactivity and weight of samples were measured using an automated gamma counter (Hidex). Data were decay corrected to time of sacrifice and calculated as the percent injected dose per gram of tissue (%ID/g) as calculated by normalizing to the total activity injected.

Formalin-fixed tissues were allowed to decay over 10 half-lives of ^89^Zr, ~ 1 month. Tissues were then embedded in OCT medium for cryo-stat preparation. Slices were cut at 6 µm thickness with every 5th section mounted for staining. Staining was performed according to the approved method for antigen retrieval by heating. The stains used were DAPI-Nuclear, AF488 (GREEN) ➔ CD8+, PE (RED) ➔ phosphor-STAT4, and APC/Cy7 (Grey/Teal) ➔ CD11b (myeloid cells). Pearson’s correlation indicated colocalization of CD8 and pSTAT.

### 2.6 Toxicology studies

#### 2.6.1 Non-GLP dose range in non-human primates

A preliminary dose-ranging study that was intended to establish the maximum tolerated dose (MTD) of SON-1010 given once, as well as the effect of repeated dosing (RD), was conducted in non-human primates (NHPs) using standard laboratory techniques (**Table 2**). A total of 28 male and female Mauritian cynomolgus macaques received SON-1010 given intravenously (IV) or subcutaneously (SC) at 31.2, 62.5, 125, or 250 µg/kg, either once on day 1 (using the full dose range), or twice on days 1 and 15 (at the lowest two doses).

**Table 2:** SON-1010 Dose Range in Non-Human Primates (non-GLP).

Cohort	Group	Test Material	Dose Route	Dose Level (mg/kg/adm)	Dose Volume (mL/kg)	Dose Concentration (mg/mL)	# of Males	# of Females
1	1	SON-1010 (hIL12- F_H_AB)	IV	0.25	5	0.05	1	1
1	2		SC	0.25	2	0.125	1	1
2	3		IV	0.125	5	0.025	1	1
2	4		SC	0.125	2	0.0625	1	1
3	5		IV	0.0625	5	0.0125	1	1
3	6		SC	0.0625	2	0.03125	1	1
4	7		IV	0.03125	5	0.00625	2	2
4	8		IV	0.0625	5	0.0125	2	2
4	9		SC	0.03125	2	0.01562	2	2
4	10		SC	0.0625	2	0.03125	2	2

Cohorts 1-3 were used to assess the Maximum Tolerated Dose (MTD) for a single IV or SC exposure to SON-1010, while Cohort 4 had a repeat IV or SC dose (RD) on days 1 and 15.

adm, administration; IV, intravenous injection; SC, subcutaneous injection.

#### 2.6.2 GLP dose range in non-human primates

In the subsequent Good Laboratory Practice (GLP) study, the safety, toxicology, and toxicokinetic (TK) attributes of SON-1010 were evaluated in 32 male and female cynomolgus macaque NHPs (**Table 3**). The dose range of 0, 15.6, 31.2, or 62.5 µg/kg given by SC injection on days 1, 15, and 29 was targeted to assess the maximum pharmacological effect, with sample size based on prior experience. Three males and 3 females were enrolled as the main group (studied for 6 weeks), with 2 additional animals of each sex at the highest dose or as controls added as a recovery group (studied for 11 weeks). The protocol and procedures involving the care and use of animals in the study were reviewed and approved by Charles River Institutional Animal Care and Use Committee (IACUC) before conduct. This study was aligned with the ICH and FDA guidelines for preclinical assessment of biopharmaceutical products.

**Table 3:** SON-1010 Dose Range in Non-Human Primates (GLP).

Group	Test Material	Dose Level (mg/kg/dose)	Dose Volume (mL/kg)	Dose Concentration (mg/mL)	Main Study		Recovery Study	
					# of Males	# of Females	# of Males	# of Females
1	Vehicle	0	2	0	3	3	2	2
2	SON-1010	0.0156	2	0.0078	3	3	0	0
3	SON-1010	0.03125	2	0.015625	3	3	0	0
4	SON-1010	0.0625	2	0.03125	3	3	2	2

Dosing occurred on Days 1, 15, and 29.

Vehicle = 200 mM Trehalose, 50 mM alanine, 25 mM Gly-Gly, 0.02% PS20, 10 µM DTPA, pH 7.1.

Following each of the three SC administrations on days 1, 15, and 29, animals were followed closely for clinical observations, body weight, and food consumption. Animals were routinely monitored ophthalmologically and by electrocardiography, along with comprehensive hematological and clinical chemistry assessments. Necropsy included gross dissection, organ weights, and histopathology. Bioanalytical samples were taken at various points during the study for cytokines, immunophenotyping, anti-drug antibody (ADA), and toxicokinetics (TK) analysis. The cytokines assessed included IFNγ, TNFα, IL-6, IL-8, IL-10, and IL-1β. ADA (IgG or IgM) was quantified in sera in two stages, starting with a screening assay followed by a confirmatory assay.

SON-1010 was quantified for TK analysis using two validated IL-12 ELISA assays (a low sensitivity assay using Thermo Fisher #88-7126-88 and a high sensitivity assay using Abcam #ab46035) to determine the concentration of SON-1010 in monkey serum. SON-1010 was spiked at 5.25 ng/mL (for the low sensitivity assay) or 1.35 ng/mL (for the high sensitivity assay) in undiluted pooled monkey serum (Charles River # CRL-PMS-013) and then aliquoted and stored for making calibration curves and QC samples. Background levels of monkey IL-12 were negligible, so were not considered to interfere with the assay. The upper and lower limits of quantitation (ULOQ and LLOQ) for the high-sensitivity assay were determined to be 135 pg/mL and 12 pg/mL, respectively and 525 pg/mL and 30 pg/mL for the low sensitivity assay, respectively. Results below the LLOQ were estimated at 12 pg/mL for graphing, while the NCA analysis treated these results as zero before dosing and as missing after dosing. Samples were diluted into the quantifiable range if the initial result was above the ULOQ. TK parameters were estimated using non-compartmental analysis (Pheonix WinNonlin).

## 3 Results

### 3.1 Selection of the F_H_AB scFv

Solid-phase panning recovered 1209 3^rd^ round binder scFv PPE’s, of which 386 (32%) were positive binders to rHSA. The positive rHSA binders were pooled and analyzed for their binding to sHSA; 371 (96%) of the clones tested bound sHSA. Out of these rHSA/sHSA positive clones, 241 (65%) also tested positive for MSA binding. The rHSA/sHSA/MSA ELISA positive clones were sequenced and 60 clones had unique V_H_ domains. Of the 60 unique V_H_ diverse clones, six were selected as primary candidates based on the prespecified criteria. The 6 clones were re-confirmed for antigen specificity by ELISA to serially diluted rHSA. All selected samples showed clear antigen concentration-dependent response curves, indicating that all candidates bound specifically to HSA.

The three-round HSA solution panning showed enrichment of phage over each previous round of selection, indicating panning success. ScFv PPE from 372 selected clones were tested in an ELISA to confirm binding to plate bound HSA, showing 300 positive scFv-PPE binders, a hit rate of 81%. Of the 300 positive HSA binders, 181 (60%) displayed cross reactivity to MSA and nine were selected as primary candidates based on the prespecified criteria.

Detection of dual signals in the FcRn competition assay indicated binding to HSA and the lack of overlapping scFv and FcRn binding domains on HSA. All selected clones from the solid and solution binding steps demonstrated binding to HSA with no effect on FcRn binding. Sequence analysis of positive HSA cross-reactive scFv clones indicated a highly diverse family of antibodies were selected, including different gene family members of V_H_ (V_H_3 and V_H_5) and V_L_ (V_L_2, -3, -5 and -9). The cross-reactive clones also displayed a high distribution of naturally occurring V-region gene families (V_H_3, V_L_2 and V_L_3). Candidates were also tested for their binding to SPARC at lower pH, to emulate the TME. A total of 15 unique clones were carefully chosen for further characterization using the previously described selection criteria.

Of these 15 clones, only one was identified from both the solution and solid phase panning methods, which was designated A10 (**Supplementary Figure 1**). Using a clone produced from the pET22b expression vector, we showed that the A10 protein purified using a preparative size exclusion column (SEC) has a K_d_ of 27.7 nM to HSA. Modifications to minimize the potential for immunogenicity resulted in the selection of clone A10m3. IL12-A10m3 produced from either HEK293T or CHO cells was fully active, both *in vitro* and in cell-based assays. IL12-A10m3 was capable of binding to mouse serum albumin, with an equilibrium K_d_ of 2.1 nM. IL12-A10m3 produced from HEK293T was also capable of stimulating human PBMC proliferation and secretion of interferon gamma (IFNγ), comparable to that of in-house produced mouse IL-12 and commercially available mouse IL-12. Thus, clone A10m3 was considered to be biologically active, sterically unhindered, and was selected as the lead F_H_AB.

### 3.2 Characterization of SON-1010

#### 3.2.1 *In vitro* characterization

##### 3.2.1.1 Testing of GMP production lots

**Figure 3:**
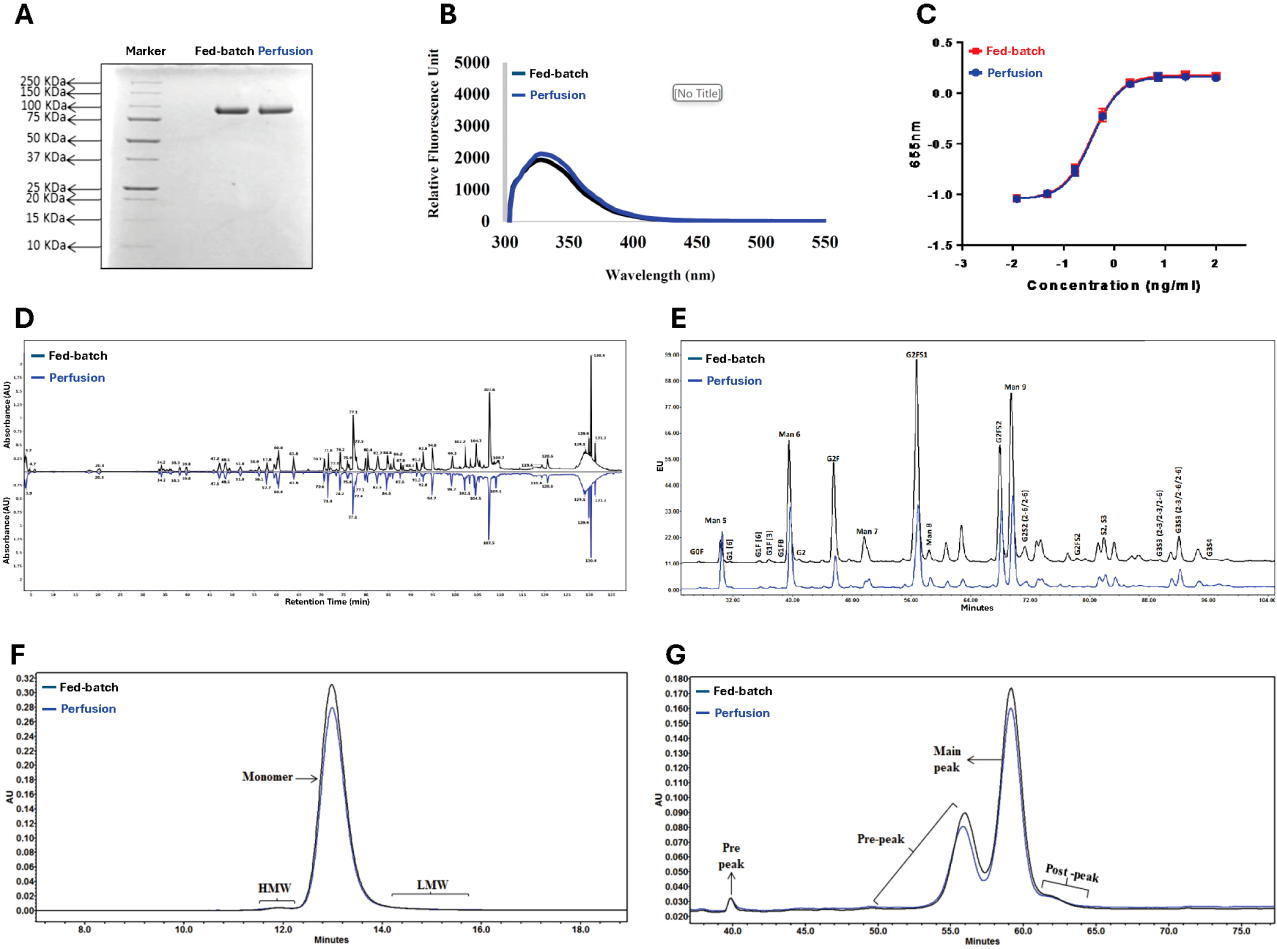
Comparison of SON-1010 DS Lots Produced by Fed-batch or Continuous Perfusion. The original GMP manufacturing process used fed-batch cultures for upstream production. This was changed to a continuous perfusion process, which increased productivity and was easier to scale up for eventual commercialization, so a series of product comparisons were performed Including: **(A)** Non-reducing SDS-PAGE, **(B)** Comparative fluorescence spectroscopy, **(C)** HEK-Blue™ IL-12 cell potency testing, **(D)** Non-reduced peptide mapping elution pattern profiling, **(E)** Glycoform analysis by HILIC, **(F)** SEC-HPLC chromatography, and **(G)** RP-HPLC chromatography. DS, drug substance; HEK, human embryonic kidney; HILIC, hydrophilic interaction chromatography; HMW, high molecular weight; LMW, low molecular weight; RP-HPLC, reverse phase-high-performance liquid chromatography; SDS-PAGE, sodium dodecyl-sulfate polyacrylamide gel electrophoresis; SEC-HPLC, size exclusion-high performance liquid chromatography.

The similar single bands observed by SDS-PAGE in the molecular range of 75-100 kDa demonstrate the comparability of the GMP fed-batch and perfusion process DS lots (**Figure 3**). The products had nearly identical fluorescence spectroscopy profiles. Both were equally potent in the HEK-Blue cell-based assay. Peptide mapping and glycoform analysis showed nearly identical patterns, confirming the lack of significant process variability. Purity was assessed by SEC-HPLC, which showed 99.1% and 98.8% monomer, respectively, while RP-HPLC showed nearly identical pre-, main-, and post-peak profiles. These assays contributed to the full proprietary set of studies that were used to show adequate control of manufacturing and readiness for clinical use, upon review by FDA.

##### 3.2.1.2 Binding studies

The initial studies done in solution showed that the identity of the molecule linked to F_H_AB does not impact affinity; the results comparing 2 different cytokines (IL-12 and GM-CSF) linked to F_H_AB had similar binding to HSA (**Table 4**). Note that the F_H_AB actually binds albumin more strongly at an acidic pH with a K_d_ of 50-200 µM.

**Table 4:** Binding Constants for SON-1010.

pH	SON-1010:HSA	GM-CSF-F_H_AB:HSA	SON-1010:HSA:FcRn	SON-1010:HSA:SPARC
**7.2**	90 nM	150 nM	35 µM	No binding
**5.8**	50 nM	50 nM	200 nM	10 nM*

*pH=6.0.

SPR was used to evaluate the more complex binding to the FcRn or SPARC (34, 39). HSA can be bound to a microchip, then various concentrations of SON-1010 can be added to determine the binding constant. The SPR response to FcRn + SON-1010 was greater than that for SON-1010 by itself, indicating formation of a ternary complex. A similar effort was conducted with SPARC, that showed tight binding with a K_d_ of 10 nM at pH 6.0 with no appreciable signal at pH 7.2. This fits with the theory that SPARC enhances internalization and transendothelial transport of albumin in endothelial cells (35). In this case, SON-1010 bound to albumin is pulled through the endothelial cells into the TME and is retained in that space by extracellular SPARC, giving the IL-12 extended time to activate the local immune response for better tumor control (**Figure 1**).

#### 3.2.2 *In vivo* characterization

##### 3.2.2.1 Initial proof of concept in the 4T1 mouse mammary tumor model

**Figure 4:**
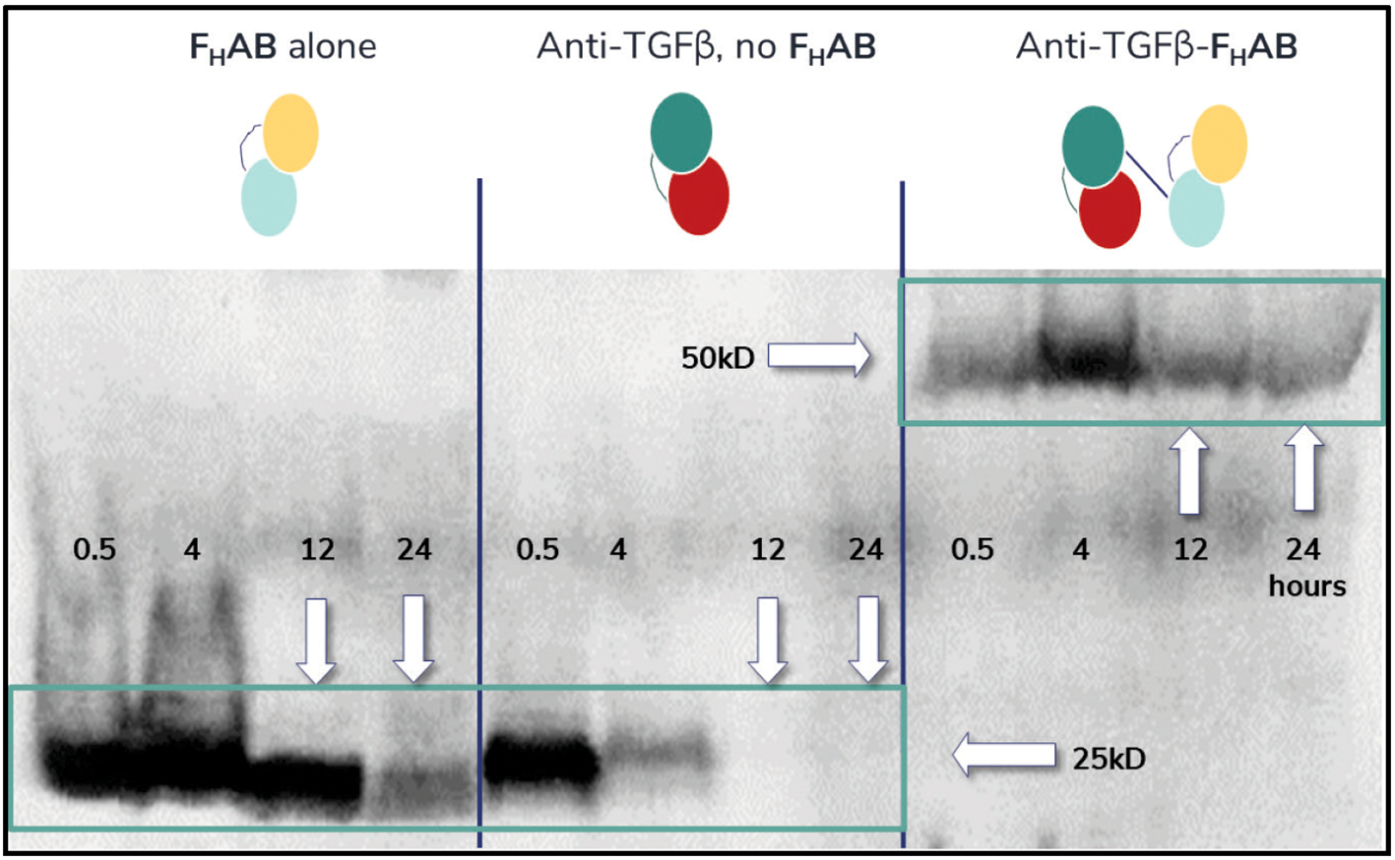
Western Blot Analysis of Mouse 4T1 Extracts. Western blot analysis of mouse 4T1 breast tumor lysates detecting the F_H_AB, anti-TGFβ, or anti-TGFβ-F_H_AB at 0.5- to 24-hours after IV injection shows accumulation. Left panel: His-tagged F_H_AB can be detected in lysates from 100 mm^3^ tumors soon after injection and at least 24 hours later. Middle panel: An anti-TGFβ scFv will bind to the TGFβ expressed by the tumor cells initially but dissipates rapidly. Right panel: When the anti-TGFβ molecule is linked to the F_H_AB, which binds to albumin *in vivo*, it can be detected in tumor lysates for at least 24 hours after dosing.

Initial pharmacokinetic work with mIL12-F_H_AB demonstrated a 4-fold improvement in half-life versus IL-12 alone, suggesting that the F_H_AB binds to albumin *in vivo*, which then associates with FcRn to increase serum half-life and slow metabolism (34). The 4T1 mouse mammary tumor model was used as an early proof-of-concept (POC) study. The isolated F_H_AB scFv efficiently targeted the implanted tumor and was present from 0.5 to 24 hours after injection (**Figure 4**). An anti-TGFβ scFv, which strongly binds to TGFβ on the tumor cell surface with a K_d_ of 10 nM, targeted the tumor as well but it was only present in lysates for up to 4 hours, suggesting that it had diffused out of the tumor at later timepoints. Linking the F_H_AB to the anti-TGFβ molecule extended the detection to at least 24 hours. The results suggest superior accumulation and retention of albumin-bound F_H_AB within the tumor, whether used alone or when linked to anti-TGFβ, and provided POC for increased immunotherapeutic efficacy of payloads linked to the F_H_AB.

##### 3.2.2.2 Comparisons of mIL-12 and mIL12-F_H_AB in the B16-F10 mouse melanoma model

The B16-F10 mouse model was first used to establish the impact of equimolar doses of the drug candidate, ranging from 4.5 µg to 30 µg mIL12-F_H_AB compared to 3 µg to 20 µg mIL-12 (the molar equivalent amount at each dose level), to study drug accumulation and its effect in controlling the growth of tumors over time. Three mice in the high-dose group were initially sacrificed at 24 hours to assess drug accumulation using a HIS-tag ELISA; greater levels of mIL12-F_H_AB were present compared to the levels of mIL-12 in all cases (**Supplementary Figure 3**). The concentrations of mIL-12-F_H_AB were increased in serum, tumor, and spleen by 128-fold, 5.6-fold, and 18-fold, respectively, over the mIL-12-dosed mice.

**Figure 5:**
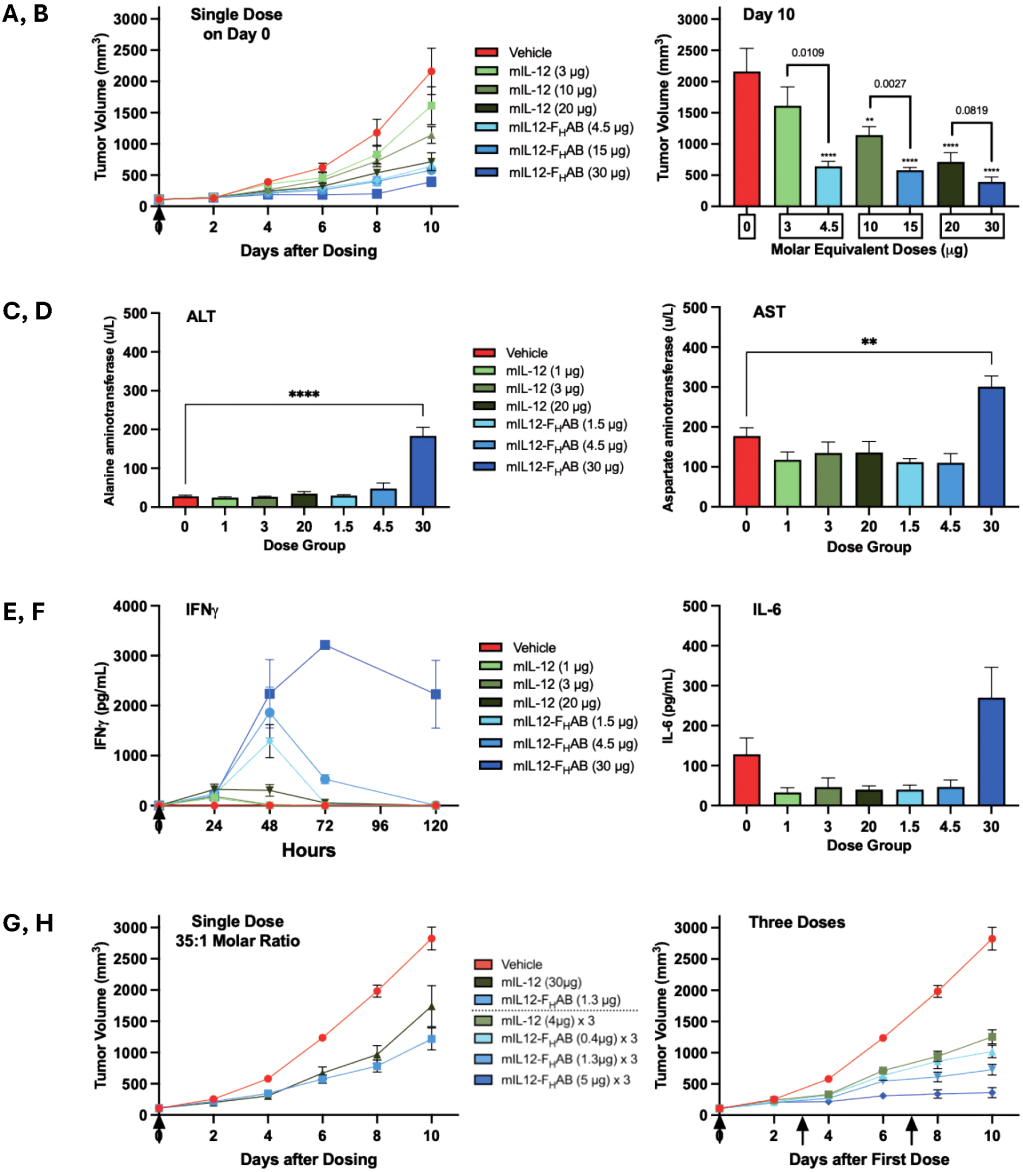
Comparison of Tumor Growth and Toxicity at Various Doses of mIL-12 or mIL12-F_H_AB. Several studies were conducted to establish the comparative effects of mIL-12 (green) versus mIL12-F_H_AB (blue) dosed once or three times (arrows). **(A)** Treatment with a single dose of mIL-12 or an equimolar amount of mIL12-F_H_AB in each paired group of 6 mice resulted in dose-dependent decreases in tumor growth over time. **(B)** On day 10, IL12-F_H_AB dosing showed further decreases in tumor volumes than mIL-12. Tumor volumes were significantly smaller in all groups except the lowest dose mIL-12; by one-way ANOVA the overall significance due to treatment was *p* < 0.0001. The significance of multiple comparisons of each group versus the vehicle control group are denoted by asterisks (** *p* < 0.01, **** *p* < 0.0001). Analyses using unpaired *t*-tests identified differences between the molar equivalent pairs; *p*-values are noted above each pair). **(C–E)**. In a similar single-dose study, transaminitis was only associated with the highest dose of mIL12-F_H_AB on Day 5 with 6 mice per group; both the ALT **(C)** and the AST **(D)** were significantly elevated five days after a dose of 30 µg, based on multiple comparisons in a one-way ANOVA (** *p* < 0.01, **** *p* < 0.0001). IFNγ **(E)** was significantly elevated and dose-dependent in all mIL12-F_H_AB groups by unpaired *t*-test comparing the low, medium, and high dose molar equivalent pairs at 72 hours (*p* < 0.05, < 0.01, and < 0.0001, respectively), while IL-6 **(F)** was only pronounced in the highest dose group. **(G)** Groups of 10 mice treated once with 35-times less mIL12-F_H_AB (based on molar equivalence) compared to the dose of mIL-12 still had a reduction in tumor growth at 6 days, and even better reduction by 10 days. **(H)** Groups of 8 mice given three doses of mIL12-F_H_AB on days 0, 3, and 7 had reduced tumor growth compared to mIL-12, even at a much lower dose equivalent. The mice given mIL12-F_H_AB at 5 µg three times showed almost no tumor growth during the 10-day study.

Tumor growth was then compared over time. After a single dose, mice receiving either mIL-12 or mIL12-F_H_AB had a decreased tumor growth rate compared to placebo (**Figure 5A**). In comparing pairs of equimolar doses of mIL-12 vs. mIL12-F_H_AB, mice dosed with the latter experienced a decreased tumor growth rate at each dose level. When assessing individual doses, a slower tumor growth rate was seen with increased doses of each compound. The tumor volume was decreased on day 10 in the treated groups compared to the placebo group (**Figure 5B**). Dosing with mIL12-F_H_AB resulted in significantly lower tumor volumes on day 10 for all but the highest dose group paired comparisons.

##### 3.2.2.3 Toxicity of mIL-12 or mIL12-F_H_AB in the B16-F10 melanoma model

The B16-F10 mouse melanoma model was used to further study the toxicity of dosing using the F_H_AB construct in a short-term study. Seven groups of 6 melanoma-bearing C57BL/6 mice/group were administered either vehicle as a placebo, different dose levels of mIL-12 (1, 3, or 20 µg), or equimolar doses of mIL12-F_H_AB (1.5, 4.5, or 30 µg). Once the tumors reached ~100 mm^3^, treatments were administered as a single IV dose on Day 0. Evaluation of tumor volumes at 5 days indicated that mIL12-F_H_AB treatment was more effective than treatment with mIL-12. Treatment with mIL12-F_H_AB at doses of 1.5 to 30 µg resulted in greater tumor reductions when compared to the molar equivalent doses of mIL-12 at 1 to 20 µg (**Supplementary Figure 4**).

In this study, the 4.5 µg dose of mIL12-F_H_AB actually showed the best reduction in tumor growth at 5 days, perhaps because the 30-µg dose led to some hepatic toxicity. The alanine aminotransferase (ALT) liver enzymes were significantly elevated at the highest level of mIL12-F_H_AB (**Figure 5C**). Aspartate aminotransferase (AST) levels showed a similar profile (**Figure 5D**). The ALT and AST values were normal at all levels of mIL-12, suggesting that the non-toxic therapeutic dose of mIL12-F_H_AB may be limited to a level of approximately 4.5 µg in mice. IFNγ was elevated in the mIL12-F_H_AB groups in proportion to dose and peaked at 48 to 72 hours after injection, with an extended time for return to baseline (**Figure 5E**). IL-6 was stable for all mice on Day 5, apart from the group with the highest level of mIL12-F_H_AB, where it was mildly elevated (**Figure 5F**).

##### 3.2.2.4 Establishing a therapeutic index for mIL12-F_H_AB in the B16-F10 melanoma model

The B16-F10 mouse melanoma model was used to compare tumor growth after a low single dose of mIL12-F_H_AB versus a 35-fold molar equivalent dose of mIL-12. Once the tumors reached ~100-150 mm^3^, groups of 10 C57BL/6 mice were administered either vehicle as a placebo, mIL-12 (30 µg as a single dose), or mIL12-F_H_AB (1.3 µg as a single dose) as a single IV dose on day 0 (**Figure 5G**).

Body weight measurements for the treated groups in this study showed changes of < 5% for all treatment groups. Tumor volume measurements showed that both mIL-12 at 30 µg and mIL12-F_H_AB at 1.3 µg showed ~50% reduction on days 2-8. By day 10, tumors in the mIL12-F_H_AB group showed ~65% lower tumor volume, while the mIL-12 group had only ~30% reduction compared to placebo. Thus, the therapeutic index for mIL12-F_H_AB was about 35-fold more effective versus free mIL-12.

With respect to toxicity, after a single dose of either compound in the B16F10 mice, transient hematological decreases were seen in total WBCs, neutrophils, and lymphocytes at Day 3 compared to the placebo group, returning to normal levels by Day 7 after dosing (**Supplementary Figure 5**). Both AST and ALT were mildly elevated transiently in the treated groups compared with the placebo mice in this study but had normalized by Day 7. IFNγ was higher with the mIL12-F_H_AB group versus mIL-12, particularly at Day 3.

##### 3.2.2.5 Study of multiple doses of mIL12-F_H_AB in the B16-F10 melanoma model

Dosing with mIL12-F_H_AB three times was then studied, along with hematologic and liver enzyme analysis for toxicity. Once the tumors reached ~100-150 mm^3^, treatments were administered as a single IV dose to 8 C57BL/6 mice per group using either vehicle as a placebo, mIL-12 (4 µg in each of 3 doses), or mIL12-F_H_AB (0.4, 1.3, or 5 µg in each of 3 doses) on days 0, 3, and 7 (**Figure 5H**).

When 3 doses were given, body weight measurements in the treated groups showed changes of < 5% for all treatment groups, except for a 7% decrease after 5 µg mIL12-F_H_AB that rebounded 2 days after the last dose. A stronger reduction in tumor volume was seen with multiple doses of mIL12-F_H_AB, along with a dose response. Note that the mIL-12 dose of 4 µg is slightly more than the molar equivalent dose of mIL12-F_H_AB at 5 µg. However, that high dose group showed a nearly flat reduction (~90%) in B16-F10 tumor volume, compared to the mIL-12 reduction of ~40%.

After 3 doses, total WBC (neutrophils + lymphocytes) showed only mild decreases in all treated groups by Days 12 and 14 (**Supplemental Figure 6**); lymphocytes showed a greater decrease than neutrophils at those timepoints. Both AST and ALT were elevated more in the placebo mice compared to all treated groups in this study. The AST with mIL12-F_H_AB was lower than with mIL-12 and the ALT showed no differences between any groups in this study. IFNγ was higher with the mIL12-F_H_AB 5 µg group versus 4 µg mIL-12 on Days 12 and 14 as well.

### 3.3 Biodistribution studies

#### 3.3.1 Tissue biodistribution by ^89^Zr Imaging and γ-Counting

SUV quantitation from imaging of select tissues shows retention and clearance primarily through the liver and kidneys after IV dosing. Image analysis indicated that the clearance of the ^89^Zr-mIL-12 was rapid, with increased levels in the liver and kidney at 3 hours, then dropping to background at the 12-hour imaging timepoint. The liver uptake of ^89^Zr-mIL12-F_H_AB at 3 hours was approximately 3-fold higher by SUV analysis than the ^89^Zr-mIL-12, and the elimination from the liver was much slower, following a steady decline over each timepoint. The ^89^Zr-mIL12-F_H_AB level and retention were increased in the kidney. Accumulation in mouse bone is a well-documented phenomenon with similar antibodies labeled with DFO chelated ^89^Zr that is caused by bone accumulation of free ^89^Zr due to its affinity for phosphates found in bone mineral deposits (40, 41). This has been observed in multiple preclinical antibody studies but has not impacted the clinical translation of this technique for ^89^Zr-antibody imaging in humans. Both groups had slower tumor growth kinetics compared to control, as in the prior studies. Measuring the accumulation of the tracer in additional tissues collected at necropsy via gamma counting confirmed the imaging data, with the earliest and highest detection levels being measured at the 6-hour timepoint. This indicates an acute accumulation in the liver that is then resolved through accumulation in the kidney. The addition of the F_H_AB to mIL-12 increased the retention across quantified tissue types. Additionally, there was a retention of mIL12-F_H_AB seen in the spleen that was not evident with the mIL-12 alone.

**Figure 6:**
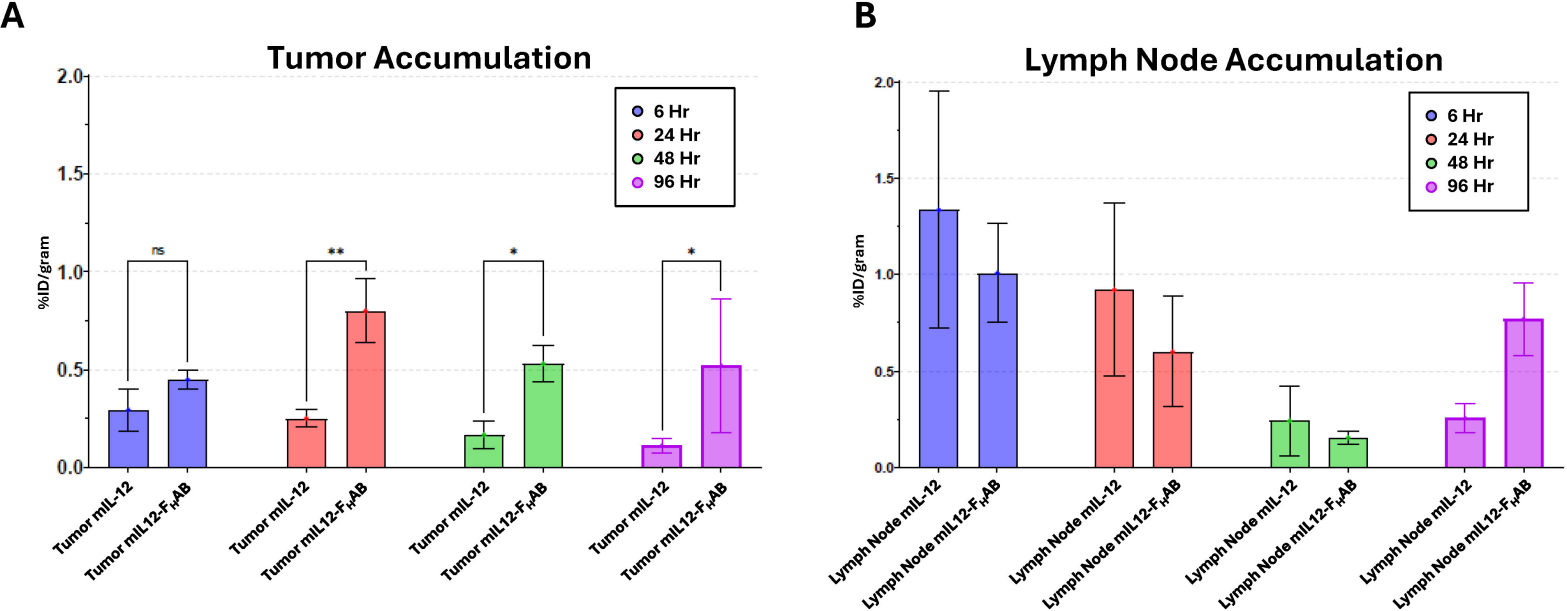
Accumulation of ^89^Zr-mIL-12 and ^89^Zr-mIL12-F_H_AB in Tumor Tissue and Inguinal Lymph Nodes. Longitudinal biodistribution studies of ^89^Zr-radiolabeled mIL-12 and mIL12-F_H_AB were performed in B16F10 tumor-bearing mice. The accumulation of ^89^Zr-radiolabeled mIL-12 or mIL12-F_H_AB was quantitated from tumors **(A)** and draining lymph node **(B)** tissue at 6-, 24-, 48- and 96-hours. Significant (two-way ANOVA; *p* < 0.05) retention of mIL12-F_H_AB occurred at 24-hours post treatment and persisted to the end of the study in comparison to mIL-12 alone. (* p < 0.05, ** p < 0.01).

Focused examination of accumulation in the tumor and draining lymph nodes indicated retention in the tumor was relatively static for both mIL-12 and mIL12-F_H_AB at each time point, with levels maintained throughout the study as determined by γ-counts (**Figure 6**). Significant retention of mIL12-F_H_AB in tumor tissue occurred at 24-hours post-treatment and persisted to the end of the study, in comparison to mIL-12 alone. Additionally, for specific γ-counts, the %ID/gram was calculated and is shown as average counts per group per time-point (**Table 5**). The average counts are calculated based on the signal per gram. The standard deviation in all of the samples is driven by the γ−counts per gram. The variability in tumor samples is due to the effect of the mIL12-F_H_AB on the tumor size, affecting the total amount of ^89^Zr/gram of tissue.

**Table 5:** Accumulation of mIL-12 vs. mIL12-F_H_AB.

		%ID/gram			
		6h	24h	48h	96h
^89^Zr−mIL−12	Heart	0.08+0.04	0.06+0.02	0.03+0.02	0.02+0.01
	Kidney	2.79 ± 0.42	5.31 ± 2.18	2.32 ± 0.43	1.96 ± 0.67
	Spleen	0.15 ± 0.08	0.10 ± 0.05	0.08 ± 0.08	0.06 ± 0.03
	Liver	40.78 ± 3.98	0.49 ± 0.24	0.44 ± 0.06	0.29 ± 0.13
	Lymph Nodes	1.34 ± 0.62	0.92 ± 0.45	0.24 ± 0.18	0.26 ± 0.08
	Tumor	0.26 ± 0.11	0.25 ± 0.05	0.17 ± 0.07	0.11 ± 0.04
^89^Zr−mIL12−F_H_AB	Heart	2.37+1.72	0.78+0.45	0.56+0.43	0.39+0.16
	Kidney	4.76 ± 1.36	3.29 ± 1.80	0.94 ± 0.50	2.37 ± 0.86
	Spleen	1.40 ± 0.39	1.65 ± 0.46	1.62 ± 1.14	1.75 ± 0.75
	Liver	20.20 ± 3.19	17.17 ± 2.52	4.62 ± 1.29	7.36 ± 1.06
	Lymph Nodes	1.01 ± 0.26	0.60 ± 0.29	0.16 ± 0.04	0.77 ± 0.19
	Tumor	0.45 ± 0.05	0.80 ± 0.16	0.53 ± 0.13	0.52 ± 0.34
	Tumor Fold-rise:	1.7	3.2	3.1	4.7

#### 3.3.2 Confocal assessment of draining lymph nodes

**Figure 7:**
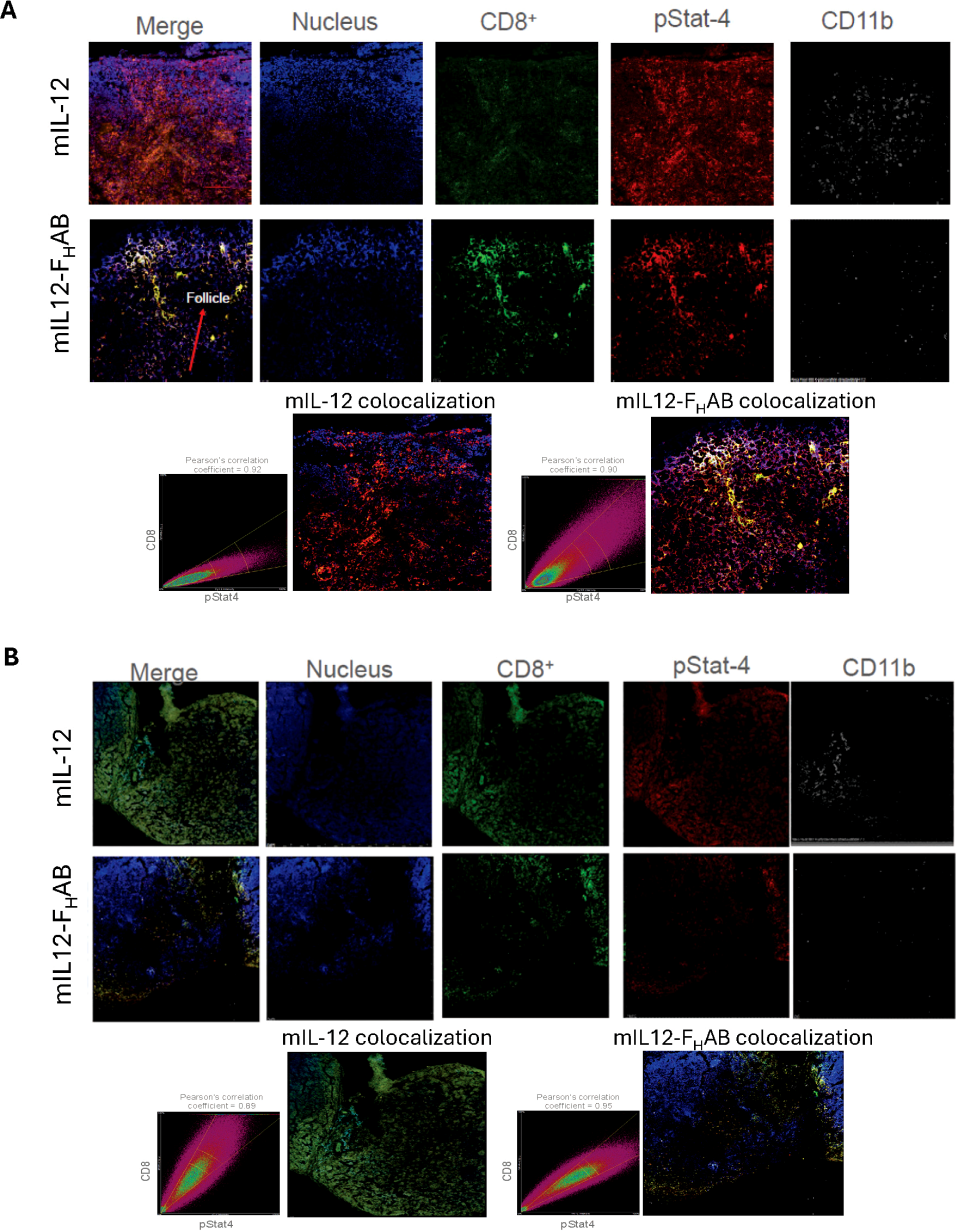
Confocal Images of Draining Lymph Nodes at 24- and 96-hours Formalin-fixed draining inguinal lymph nodes (adjacent to the tumor implant) were allowed to decay after dosing with ^89^Zr-radiolabeled mIL-12 or mIL12-F_H_AB over 10 half-lives, then embedded in OCT medium and sliced at 6 µm thickness. Confocal microscopic analysis of the sections was done after staining as indicated. The time-points of **(A)** 24- and **(B)** 96-hours were selected for an acute and prolonged response, respectively. Images were acquired in the z-dimension to create a maximum-intensity projected composite image of an average of 15 slices taken at 1-micron increments. Colocalization of CD8 (green) and phosphor-STAT4 (red) was assessed using Pearson’s correlation coefficient, which is an assessment of the coincidence of two fluorophores existing in the same voxel (volumed pixel), at each time point. Nuclear staining (blue) and macrophages, represented by CD11b staining (grey), are also represented. Pearson’s correlation indicated colocalization of CD8 and pStat.

An assessment of CD8 T cells after stimulation by mIL-12 or mIL12-F_H_AB was performed at 24- and 96-hours after dosing using confocal microscopy with tissue from the draining inguinal lymph node adjacent to the tumor implant (**Figure 7**). This was the most proximal site for lymphocyte trafficking and a likely site for activation and maturation of T-cells. Images of lymph nodes were acquired in the z-dimension to create a maximum-intensity projected composite image using an average of 15 slices taken at 1-micron increments. CD8 T cells (green), phospho-STAT4 staining indicating cytokine signaling (red), nuclear staining (blue), and macrophages shown by CD11b staining (grey) are each presented individually. Analysis of the split channel images from animals treated with mIL12-F_H_AB shows a reduction in the CD11b-positive cells at 24-hours in the lymph node, corresponding with an increased abundance of CD8-positive cells in what appears to be the follicular zone of the node. This is not evident in the mIL-12 treated animals.

Colocalization of CD8 and pSTAT4 was assessed using Pearson’s correlation coefficient. At 24-hours after mIL-12 treatment, the pSTAT4 mean fluorescence intensity (MFI) is more prevalent in the field, while the CD8 staining intensity was not significantly above autofluorescence levels. At the same time after mIL12-F_H_AB treatment, there is distinct CD8 staining that colocalizes with distinct pSTAT4 staining at nearly a 1:1 ratio, associated with a Pearson’s correlation coefficient value of 0.90. Analysis at 96-hours showed the effect of retention of mIL12-F_H_AB in the lymph node. The physiological activity of T-cells in the lymph nodes was maintained, along with continued colocalization of CD8 and pSTAT4 (Pearson’s correlation coefficient = 0.95). The abundance of the CD8^+^ T-cells is reduced at 96 hours in the mIL-12-F_H_AB treated animals, as they are likely trafficking back to the tumor.

The animals treated with mIL-12 again demonstrate either non-active T-cells, with the CD8^+^ staining being predominant but not colocalized with pSTAT4, or weak CD8 activation. This is also consistent with the biodistribution data (**Figure 6**). Additionally, an examination of the CD11b staining as a macrophage marker shows consistent reduction in fluorescence at both 24- and 96-hours in mIL12-F_H_AB treated animals, when compared to mIL-12 alone. This is consistent with the previous finding that elevated IL-12 activity correlates with a reduction of myeloid cells (13). Taken together, the confocal imaging data is supportive of a functional accumulation of mIL12-F_H_AB in the lymph node, resulting in activation of CD8 T cells and a reduction of myeloid cells, likely promoting an antitumor phenotype.

### 3.4 Toxicology studies

#### 3.4.1 SON-1010 non-GLP dose range in non-human primates

IL12-F_H_AB-related clinical observations were observed in both males and females in both MTD and RD groups. In the majority of MTD animals, clinical observations included hunched posture, mild to moderate dehydration, decreased activity, soft or liquid feces, mild, intermitted tremors and reduced appetite. In the RD animals, test article-related clinical symptoms included hunched posture, slight to moderate soft or liquid feces, reduced appetite, and mild intermittent tremors.

Hematology changes related to IL12-F_H_AB initially included an overall decrease in all MTD groups in white blood cells, neutrophils, lymphocytes and monocytes, eosinophils, basophils, and platelets. The decrease was observed on day 3 and 7, followed by the recovery of an overall increase reaching the pretest values on day 14 and/or 21. The changes were not dose or route of administration dependent. Similar findings occurred in the RD animals, with recovery starting on day 10. On day 17 (48 hours post second dose) a slight decrease in white blood cells and lymphocytes was noted in all groups compared to day 10, followed by recovery on day 21. The flow cytometry data demonstrated that dosing IL12-F_H_AB in all animals resulted in the margination of peripheral lymphocytes causing a decrease of peripheral absolute total T-cells, T-helper cells, cytotoxic T cells, and NK cells for all animals by day 3. All T-cell types recovered back to baseline at a greater extent than NK cells and B cells in all groups.

The clinical chemistry changes related to IL12-F_H_AB administration included mildly increased values for aspartate aminotransferase on day 7, and total bilirubin on day 3 in a majority of animals in the MTD groups. In the RD groups, an increased level of aspartate aminotransferase on day 7 was observed. The total bilirubin level was increased in males from the lower dose group. The level of aspartate aminotransferase and total bilirubin gradually returned to prestudy values by day 14 and/or 21. The cytokine data supports IL12-F_H_AB test article-related effects on IFNγ and uncertain effects on IL-6, IL12(p40) and TNF-α. Although there is test article effect on IFNγ, this effect was not shown to be a dose-dependent response.

Both the male and female dosed at 250 µg/kg by IV administration were euthanized on day 10 due to severe clinical and veterinary observations, including moderate dehydration, decreased activity, hunched posture, cold to touch, pallor skin, bloody liquid diarrhea and lack of interest in food. In addition, both animals lost 7% body weight on day 8, relative to pretest. A macroscopic finding of spleen and lymph node enlargement were noted in both animals. The hematology changes included decreases in white blood cells, neutrophils, lymphocytes, monocytes, eosinophils, basophils, large unstained cells, platelets and red blood cells mass (red blood cells, hemoglobin, hematocrit) starting on Day 3 and 7 and continued to decrease on Day 7. On Day 10, the levels of white blood cells, neutrophils and lymphocytes returned to values that were close to those values on Day -1. The clinical chemistry changes included an increase in aspartate aminotransferase, triglycerides and globulin, and decrease in phosphorus and albumin. Necropsy revealed an adrenal grand enlargement, small thymus, and dark foci on the urinary bladder in the female. None of the animals dosed SC at that dose needed to be prematurely euthanized.

In conclusion, for the MTD groups in this preliminary dose-finding study, a single SC administration of IL12-F_H_AB was generally tolerated in cynomolgus monkeys up to 250 µg/kg. Single IV administration of IL12-F_H_AB was tolerated up to 125 µg/kg. IL12-F_H_AB-related changes in clinical observations, body weight loss, clinical pathology, cytokines and immunophenotyping occurred. Most parameters recovered to pre-study values by the end of the 3-week observation period. In the RD groups, IL12-F_H_AB administrated by SC and IV injection was tolerated up to 62.5 µg/kg/adm following administration on days 1 and 15. IL12-F_H_AB-related changes in clinical observation, body weight, clinical pathology, cytokines and immunophenotyping were observed. Recovery to pre-study values by the end of the study was also observed. Test article-related macroscopic findings and organ weights were observed at ≥ 31.2 µg/kg/adm.

#### 3.4.2 SON-1010 GLP repeat dose toxicity in non-human primates

The second NHP study was done under GLP conditions and all animals survived until the scheduled euthanasia. Administration of SON-1010 to monkeys by SC injection on days 1, 15, and 29 at 15.6, 31.2, and 62.5 µg/kg/dose resulted in minor body weight changes (less than 10%) and clinical observations that included hunched posture, decreased activity, slight to moderate tremors, and reduced appetite, with fever being observed in only one high dose group female. All SON-1010-related clinical observations resolved before the recovery period and were not considered to be adverse. No SON-1010-related effects were observed upon ophthalmic examinations or electrocardiography evaluations.

Administration of SON-1010 resulted in transient changes in hematology (**Supplementary Table 1**) and clinical chemistry (**Supplementary Table 2**) parameters, starting at the lowest dose, but there were no drug-related changes in coagulation or urinalysis. SON-1010-related hematologic changes were noted from day 8 with resolution by day 21 and were up to moderate in magnitude. These changes consisted of decreased red blood cell mass, an initial decrease followed by an increase in reticulocyte count, increased red cell distribution width and platelet count, and decreased neutrophils with morphological findings suggestive of accelerated maturation, along with changes in monocyte, lymphocyte, eosinophil and white blood cell counts. Clinical chemistry changes were noted at days 8 and/or 15 and were of minimal to mild magnitude. These changes included decreased cholesterol, albumin to globulin ratio, calcium, phosphorus, sodium, and chloride, and increased globulin and triglyceride. These changes were not observed at days 43 and/or 71, indicating recovery. The SON-1010-related changes in clinical pathology parameters were considered non-adverse based on the small magnitude, transient response, and/or the lack of histopathology correlates. Administration of SON-1010 resulted in no organ weight, gross, or microscopic changes at any dose level on days 38 or 71.

In this GLP toxicology study, administration of SON-1010 by three SC injections was tolerated in monkeys up to 62.5 µg/kg/dose. Hematological changes were observed in red cells, reticulocytes, and platelets, and in neutrophils suggestive of accelerated maturation, along with changes in monocytes, lymphocytes, eosinophils and white blood cell counts. In clinical chemistry, minimal changes were observed that included decreased cholesterol, albumin to globulin ratio, calcium, phosphorus, sodium and chloride and increased globulin and triglyceride. These changes were not observed on days 43 and/or 71, indicating recovery.

The concentration of SON-1010 was measured with the dose on days 1 and 29 (**Supplementary Figure 7**) and PK was determined by noncompartmental analysis (**Supplementary Table 3**). Nearly all of the test animals in groups 2, 3 and 4 demonstrated a much lower *Cmax* for the repeat dose (Day 29) with multiple samples below the lower limit of quantitation (BLQ). It is likely that this is due to the development of anti-SON-1010 IgG antibodies as ADAs were strongly induced in most of the test animals injected with SON-1010. Allometric scaling was employed to estimate human predicted values for clearance and the central volume of distribution using single species scaling for SON-1010 based on PK values from the nonhuman primate GLP toxicology study. An exponent for clearance of 0.85 for large protein molecules (42) and an exponent of 1 for the volume of distribution were selected based on the molecular size of SON-1010. As a validation of the methodology, human clearance was also calculated by a single species scaling method (43). The PK predicted for humans was CL=184.1 mL/h, V_d_=13,867 mL, t_½_=52.2 h (**Supplementary Figure 8**).

Dose-dependent reductions in NK cells were present for all SON-1010-dosed groups, with the greatest reductions evident for most of Group 4 (62.5 µg/kg) dosed animals after all three doses. Most animals in Groups 2 through 4 recovered toward baseline values prior to the second dose on day 15. Minor, transient, and variable reductions in B- and T-lymphocyte populations were present after the initial dose of SON-1010, with values generally returning to baseline by day 15. The cytokine data supports SON-1010-related effects on IFNγ and uncertain effects on IL-12(p40) and TNFα. There was an IFNγ response for all animals in Groups 2 through 4 at the Day 4 time point. Response continued through Day 7 for many animals and two animals in Group 4 had an additional response on Day 18 (3 days post the second dose). There were no effects for IL-6, IL-8. IL-10 or IL-1β. Development of neutralizing ADA to SON-1010 (**Supplementary Figure 9**) by at least day 38 likely limited the absolute exposure of Groups 2 through 4 to repeated doses of SON-1010. However, based on these results, the no-observed-adverse-effect level (NOAEL) in male and female cynomolgus monkeys following repeated SC administration of SON-1010 was 62.5 µg/kg/dose.

## 4 Discussion

Activating the immune system to destroy cancer cells has been of considerable interest for several decades but there are a number of factors that have limited interleukin efficacy in the clinic. Improved cancer immunotherapeutics will require an increase in systemic PK, immune cell recruitment and infiltration, and tumor accumulation and retention time to achieve a high degree of efficacy. Strategies to address these issues have included structure-based cytokine engineering by PEG or attachment to Fc or IgG’s as fusion proteins (44). These constructs can increase the PK half-life but lack the ability for tumor targeting and enhanced penetration and retention. The combination of tumor targeting and an increased half-life can improve efficacy, while reducing toxicity through dose-sparing and/or reduced dosing intervals. Recombinant IFNα and IL-2 are the only cytokines that have been approved in the United States for the treatment of cancer, even though their efficacy was modest.

Other recombinant interleukin and cytokine strategies have had limited success in clinical trials, despite the ability of several of these therapeutic candidates to regulate both the innate and adaptive immune systems in ways that can lead to tumor cell death in preclinical models. This is due in part to the small molecular weight of this class of proteins (<50 kDa), causing rapid renal elimination, along with the lack of tumor targeting. Approaches to improve the serum half-life of these small proteins include the creation of various formulations on the surface of or within nanoparticles. Albumin has also been exploited as a carrier for small therapeutic proteins e.g., interferon-α has been linked to albumin directly to make albinterferon alfa-2B (45). A potential advantage to the use of albumin as a drug carrier is that albumin increases serum half-life due to its larger molecular weight (> 60 kDa) and it is known to accumulate in inflamed and angiogenic tissues, such as the TME (19, 21).

Building on the concept of linking interleukins to albumin constructs to enhance the PK, we screened for and developed the F_H_AB, a scFv that binds human albumin and can be linked to one or two interleukin payloads. The small size of the scFv (26 kDa) provides an advantage over whole antibodies (~150 kDa), allowing for more rapid penetration into tissues and tumors (46). Our F_H_AB binds to albumin in the serum, which can transport the F_H_AB-albumin complex to interact with FcRn and GP60 receptors that are often upregulated in tumor endothelium.

Once the F_H_AB-albumin complex is targeted and migrates across the endothelium, the complex is able to bind tightly to SPARC in the acidic TME, thus enhancing retention and duration of activation of local immune cells in solid tumors. The F_H_AB domain linked to immune modulators can target both tumors and lymph nodes while increasing retention time, which leads to enhanced efficacy. We set out to develop this relatively small non-immunogenic cytokine carrier that could bind to albumin rather than being linked, which was designed to do this at both physiologic and acidic pH but would not interfere with its normal physiology and the ability of albumin to target the TME.

### 4.1 Generation of the best F_H_AB clone for further development

Solid-phase panning is the most straightforward method of phage display screening and is widely used for its simplicity, usually resulting in the capture of phage with varying affinities (46). The method starts with passive adsorption of the target protein onto plastic, which occurs through electrostatic, hydrophobic, and hydrophilic interactions. An antibody phage library is added to plastic wells; the library we used was derived from healthy human volunteers and had high sequence diversity to enhance the ability to find an appropriate clone. Following binding, several wash steps were incorporated to remove non-specific bound phage. Phage eluates were neutralized and utilized in subsequent rounds of panning for further enrichment of scFv clones with higher affinity and broader sequence diversity.

In contrast, solution-based panning is a more labor-intensive method that allows for a more stringent selection process with less antigen. The method traditionally results in the capture of higher affinity phage-displayed scFv, and in this case targeted rHSA covalently linked to biotin. Albumin-scFv-phage complexes were captured via streptavidin-coated magnetic beads. Because the reaction is performed in solution, phage binding to the target rHSA in its native configuration is an independent event where binding is driven by antigen concentration. The number of scFv bound to antigen is largely dependent upon the K_d_ between the displayed scFv and antigen. As such, at low concentrations of antigen at or near the K_d_, phage expressing tighter binding scFv’s will bind proportionally more antigen molecules than phage bearing weaker binding scFv’s. Therefore, at low target concentrations, more phage bearing higher affinity scFv will be captured over those bearing weaker binding scFv. The captured rHSA-scFv-phage complexes were extensively washed to remove non-specific binders. As in solid phage panning, captured phage were eluted from the antigen-scFv complex, neutralized and rescued for subsequent rounds of selection.

A series of criteria were used to select the best candidates after several rounds of concentration with each panning method. Cross-reactivity to human, mouse, and eventually to cynomolgus monkey albumin was critical to establish a path to preclinical and subsequent clinical development. Strong binding at low pH allows accumulation in an acidic environment, which is the key for efficient tumor targeting (19). The clones had to be screened for high expression level as well, to ensure eventual manufacturability. Preserving the extended PK was assured by checking for the lack of competition with binding to the FcRn. Several lead candidates were eventually chosen after checking for HSA-specific binding in whole blood and extended human serum stability. The only clone that was identified by both panning methods passed through each screen and was evaluated for its potential for immunogenicity. A single mutation was made that deleted several epitopes that were predicted to have moderate and/or high affinity. The clone with the lowest K_d_ was selected as the lead candidate for development.

The manufacturing approach was initially developed using a simple fed-batch process to eventually produce fully characterized GMP material. While this was adequate for the GLP study and the first clinical trial, scale-up and commercialization usually requires a more sophisticated approach. We developed a process that uses continuous perfusion for upstream production, along with continuous harvesting and purification in the downstream process for IL12-F_H_AB. This enabled the maintenance of key nutrients for process optimization and efficient separation of the product from long-term exposure to proteolytic enzymes and other detrimental metabolites. Comparability was established with extensive testing and characterization, as shown in **Figure 3**. Given that the material from the two manufacturers appeared and behaved nearly identically, there was a seamless introduction of the new material into the next clinical trial.

### 4.2 Preclinical characterization of the F_H_AB

Initial work utilizing the F_H_AB platform demonstrated a much longer half-life of domains fused to the scFv protein, suggesting that the F_H_AB binds to albumin. It then associates with FcRn systemically and in tumor tissue, more specifically to GP60, and then binds tightly to SPARC. Binding to SPARC has a K_d_ of 10 nM at pH 6.0, commonly found in the TME, which accounts for the targeting to that space (19, 47). The construct was retained in mouse 4T1 tumors that overexpress TGFß, as shown with anti-TGFß linked to the F_H_AB. Increased time within the tumor implies a potential for increased immunotherapeutic efficacy of payloads that are linked to the F_H_AB. While the SPARC level is relatively high in breast cancers, tumor morphology, density, and size, dose timing, number of doses, etc. are all important for drug retention as well. We use relative SPARC expression as one of the ways to select tumor indications for studies in humans.

Building upon this finding in the 4T1 model, the anti-tumor activity of mIL12-F_H_AB was evaluated over longer timeframes in a melanoma model. When mIL-12 was linked to F_H_AB, it was found to be more effective in reducing tumor volumes and extending tumor survivability than mIL-12 alone in a dose-dependent fashion in B16-F10 tumor-bearing mice, compared to placebo. The mIL12-F_H_AB anti-tumor activity was markedly enhanced, which was likely a result of the extension of half-life by linking the cytokine to the F_H_AB domain with subsequent binding to albumin. The pharmacodynamic and toxicological effects of mIL12-F_H_AB and mIL12 in mice suggest that while the therapeutic dose may be limited to a level of < 30 µg in mice, B16-F10 tumors are well controlled in that range. Early indicators of the potential for toxicity can be a guide to the surveillance of systemic inflammation. Liver enzymes were well managed and only present when the mIL12-F_H_AB doses substantially exceeded those likely to be employed in the clinic. Transient neutropenia and lymphopenia are known phenomena after dosing with recombinant hIL-12 dosing. Similar cell kinetics were seen in the studies of hIL-12 in healthy volunteers (48). The rapid recovery of cell counts suggests a margination phenomenon may be occurring, as opposed to cell destruction, as the recovery is far too rapid for new cell production.

Further work with the B16-F10 mouse model demonstrated an improved therapeutic index when comparing doses of the murine analog of SON-1010 with mIL-12 alone after a single dose that was 35 times the molar equivalent, pointing to the potential for improved efficacy with reduced toxicity, using lower doses of a compound that has an extended half-life. The further improvement in tumor control with multiple doses in this rapidly-growing B16-F10 tumor model may support the utilization of a repeat-dose regimen in humans as well.

Biodistribution was studied after checking to ensure that radiolabeling would not disrupt IL-12 or albumin-binding functionality of the F_H_AB. The tissue distribution by imaging indicated that the clearance of the ^89^Zr-mIL-12 was rapid, with detectable levels in the liver and kidney at 3 hours, then dropping to baseline at the 12-hour imaging interval. ^89^Zr-mIL12-F_H_AB detection in the same tissues indicated that the addition of the F_H_AB increased the retention time of mIL-12, but with controlled clearance through the liver and kidneys. Comparison of tumor and inguinal lymph node accumulation between mIL-12 and mIL12-F_H_AB showed significant 3- to 5-fold accumulation of the latter in the tumor and this significance was maintained for at least 96 hours. While the inguinal lymph node did not show statistically significant differences, by 96 hours there was a 3-fold increase in mIL12-F_H_AB compared to mIL-12.

The confocal images of draining lymph nodes adjacent to the tumor at 24- and 96-hours confirmed the late time-point activity of the T cells in the animals treated with mIL12-F_H_AB due to colocalization of CD8 and pSTAT4. The abundance of these CD8^+^ T-cells was reduced, as they are likely trafficking back to the tumor. Additionally, examination of CD11b staining as a macrophage marker shows consistent reduction in fluorescence at both 24- and 96-hours after mIL12-F_H_AB treatment when compared to mIL-12 alone. This is consistent with the literature indicating that elevated mIL-12 correlates to a reduction of M2 myeloid cells (12). Taken together, the confocal imaging is supportive of a functional accumulation of mIL12-F_H_AB in the lymph node, resulting in activation of CD8 T-cells and a reduction of myeloid cells, likely promoting an antitumor phenotype.

The final dataset needed before going into clinical development is the assessment of toxicity using the actual therapeutic candidate and the expected route of injection. The safety of SON-1010 was studied in cynomolgus monkeys, as this is the most relevant model for human safety and the F_H_AB had been shown to bind to CySA. An ADA response following exposure to human cytokines is a common finding in NHPs (49), however, an on-target pharmacological response was also demonstrated by an increase in IFNγ after injection of hIL-12 (50). The initial safety study typically pushes the dose to toxicity in single and multiple dose formats to establish the MTD and a NOAEL based on the maximal pharmacological effect, as dose-limiting toxicity of recombinant cytokines is most often attributed to exacerbated on-target pharmacology. While higher doses caused some toxicity, the dose of 62.5 µg/kg/dose was well-tolerated and is over 50-fold the expected maximal human dose. This was confirmed in the second study, which was done under GLP conditions with SC injection. The clinical signs at that dose were not considered to be adverse. Hematologic and clinical chemistry changes were consistent with the human experience with rIL-12 (17) and resolved during recovery. In addition, while there was a transient increase in IFNγ, there was no suggestion of cytokine release syndrome. These findings help to guide the expected adverse events with SON-1010 in clinical studies.

## 5 Conclusion

The F_H_AB domain is a platform that can deliver immunomodulators in either a mono- or bifunctional format. We have exploited the tumor-targeting properties of HSA to enhance the potential therapeutic benefits of interleukins and can dramatically extended their half-life. This F_H_AB platform provides an improved pharmacokinetic profile, benefits from albumin binding of over-expressed FcRn, GP60, and SPARC, incorporates a dose-sparing effect that further decreases the toxicity risk, and results in a broader therapeutic index for rIL-12. SON-1010 represents a novel approach that may finally help realize the potential for rIL-12 to be dosed safely and effectively in cancer treatment. This is currently being addressed in the clinic both as monotherapy (28) and in combination with atezolizumab (29).

## Data Availability

The original contributions presented in the study are included in the article/**Supplementary Material**. Further inquiries can be directed to the corresponding author/s.
