# Supplementary material for: SON-1010: an albumin-binding IL-12 fusion protein that improves cytokine half-life, targets tumors, and enhances therapeutic efficacy
Source: Front Immunol. 2024 Dec 4;15:1493257. doi: 10.3389/fimmu.2024.1493257 (PMC11652653; doi:10.3389/fimmu.2024.1493257)
Supplement: Supplementary file 1 [file DataSheet1.pdf]

Supplementary Material

Supplemental Figure 1: Selection of F_HAB Leads – Summary After Re-ranking

A: Final F_HAB Leads

Lower number = better	Expression			Immuno-genicity	affinity for HSA	affinity for MSA
Clone	SDS (Soluble fraction)	Final SEC yield (soluble fraction)	ELISA (soluble fraction)	Predicted Epitopes	K_d by Octet	K_d by Octet
A6m	2	2	1	1	1 (1-3nM)	2 (2-27nM)
A9m	3	3	3	2	2 (4-12nM)	1 (7-12nM)
A10 Hit by both solid & solution panning	1	1	2	3 (unmodified) 1 (modified)	3 (~27nM)	3 (~21nM)

B: De-immunization of F_HAB Clone A10 and Results of an Antitope and TCED Analysis

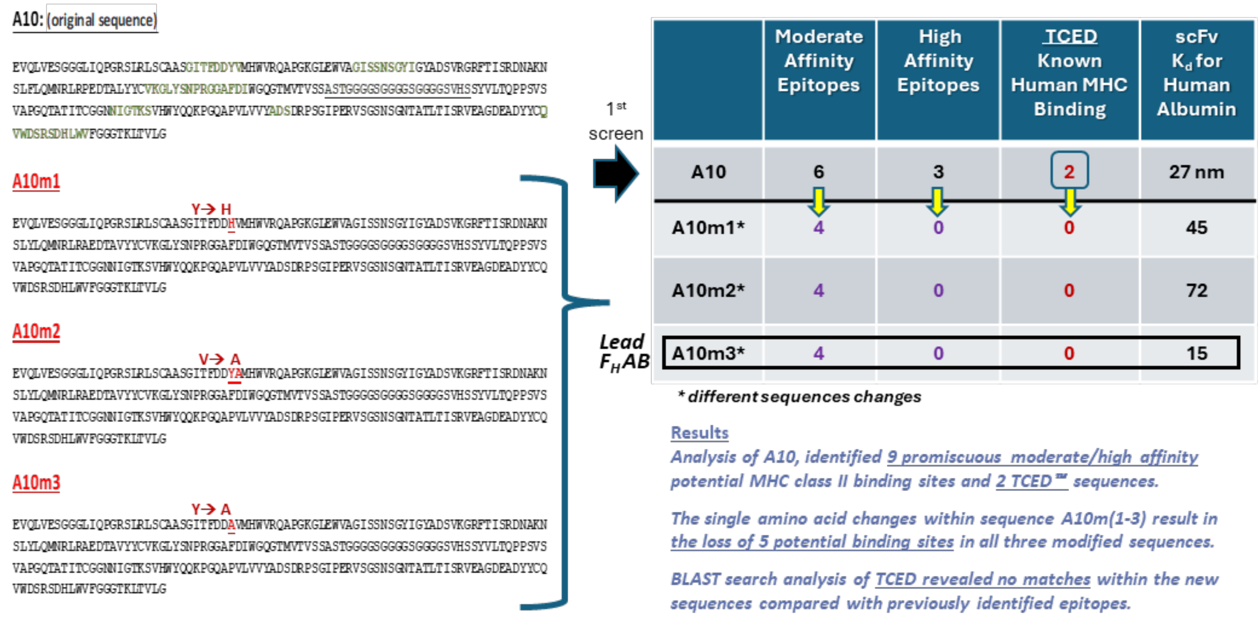

Solid- and solution-based panning led to the identification of 15 unique phage clones that did not compete with FcRn binding to albumin. After binding to HSA was confirmed by Octet, these were sequenced and run through several assays described in Section 2.1 to identify clones that bound at high affinity at both acidic and neutral pH. Clone A10, which was the only clone that was identified by both panning techniques, also gave the highest production yield. CD4+ T-cell binding sites that were predicted to be immunogenic were identified. Several mutations were studied for continued binding to HSA. Clone A10m3 was selected based on its high production yields, minimally predicted immunogenicity, and low Kd for HSA.

Supplemental Figure 2: Cytokine, Linker, and F_HAB Sequence

A

Mouse IL12-F_HAB (mIL12–A10m3) Amino Acid Sequence (799 aa):

MWELEKDVYVVEVDWTPDAPGETVNLTCDTPEEDDITWTSDQRHGVIGSGKTLTITVKEFLDAGQYTCHK
GGETLSHSHLLLHKKENGIWSTEILKNFKNKTFLKCEAPNYSGRFTCSWLVQRNMDLKFNIKSSSSSPDS
RAVTCGMASLSAEKVTLDQRDYEKYSVSCQEDVTCPTAEETLPIELALEARQQNKYENYSTSFFIRDIIK
PDPPKNLQMKPLKNSQVEVSWEYPDSWSTPHSYFSLKFFVRIQRKKEKMKETEEGCNQKGAFLVEKTSTE
VQCKGGNVCVQAQDRYYNSSCSKWACVPCRVRSGGGGSGGGGSGGGGSRVIPVSGPARCLSQSRNLLKTT
DDMVKTAREKLKHYSCTAEDIDHEDITRDQTSTLKTCLPLELHKNESCLATRETSSTTRGSCLPPQKTSL
MMTLCLGSIYEDLKMYQTEFQAINAALQNHNHQQIILDKGMLVAIDELMQSLNHNGETLRQKPPVGEADP
YRVKMKLCILLHAFSTRVVTINRVMGYLSSAGGGGSGGGGSGGGGSGGGGSGGGGSEVQLVESGGGLIQP
GRSLRLSCAASGITFDDAVMHWVRQAPGKGLEWVAGISSNSGYIGYADSVKGRFTISRDNAKNSLYLQMN
RLRAEDTAVYYCVKGLYSNPRGGAFDIWGQGTMVTVSSASTGGGGSGGGGSGGGGSVHSSYVLTQPPSVS
VAPGQTATITCGGNNIGTKSVHWYQQKPGQAPVLVVYADSDRPSGIPERVSGSNSGNTATLTISRVEAGD
EADYYCQVWDSRSDHLWVFGGGTKLTVLG

. . . .

B

Human IL12-F_HAB (hIL12–A10m3) Amino Acid Sequence (788 aa):

IWELKKDVYVVELDWYPDAPGEMVVLTCDTPEEDGITWTLDQSSEVLGSGKTLTIQVKEFGDAGQYTCHK
GGEVLSHSLLLLHKKEDGIWSTDILKDQKEPKNKTFLRCEAKNYSGRFTCWWLTTISTDLTFSVKSSRGS
SDPQGVTCGAATLSAERVRGDNKEYEYSVECQEDSACPAAEESLPIEVMVDAVHKLKYENYTSSFFIRDI
IKPDPPKNLQLKPLKNSRQVEVSWEYPDTWSTPHSYFSLTFCVQVQGKSKREKKDRVFTDKTSATVICRK
NASISVRAQDRYYSSSWSEWASVPCSGGGGGGSRNLPVATPDPGMFPCLHHSQNLLRAVSNMLQKARQTL
EFYPCTSEEIDHEDITKDKTSTVEACLPLELTKNESCLNSRETSFITNGSCLASRKTSFMMALCLSSIYE
DLKMYQVEFKTMNAKLLMDPKRQIFLDQNMLAVIDELMQALNFNSETVPQKSSLEEPDFYKTKIKLCILL
HAFRIRAVTIDRVTSYLNASGGGGSGGGGSGGGGSGGGGSGGGGSEVQLVESGGGLIQPGRSLRLSCAAS
GITFDDAVMHWVRQAPGKGLEWVAGISSNSGYIGYADSVKGRFTISRDNAKNSLYLQMNRLRAEDTAVYY
CVKGLYSNPRGGAFDIWGQGTMVTVSSASTGGGGSGGGGSGGGGSVHSSYVLTQPPSVSVAPGQTATITC
GGNNIGTKSVHWYQQKPGQAPVLVVYADSDRPSGIPERVSGSNSGNTATLTISRVEAGDEADYYCQVWDS
RSDHLWVFGGGTKLTVLG

Final protein sequence of the native **(A)** mouse or **(B)** human single-chain IL-12 linked to the scFv sequence selected to be the F_HAB domain. The p40 subunit of IL-12 is shown in blue text, the p35 subunit in purple text, the F_HAB sequence in red text, and the linkers are underlined and highlighted in yellow. A dot is shown at every 10th symbol to help with counting.

The sequences are published as Figure 20 in US Patent: **CINI, J. K. & HUANG, H**. 2019. *Albumin-binding Domain Fusion Proteins*. US 2019/0016793 Al patent application 62/459,975. Jan. 17, 2019. Both sequences have been submitted to GenBank.

Supplemental Figure 3: ELISA Analysis of Serum, Spleen, and Tumor mIL12 at 24 hours Post Dosing

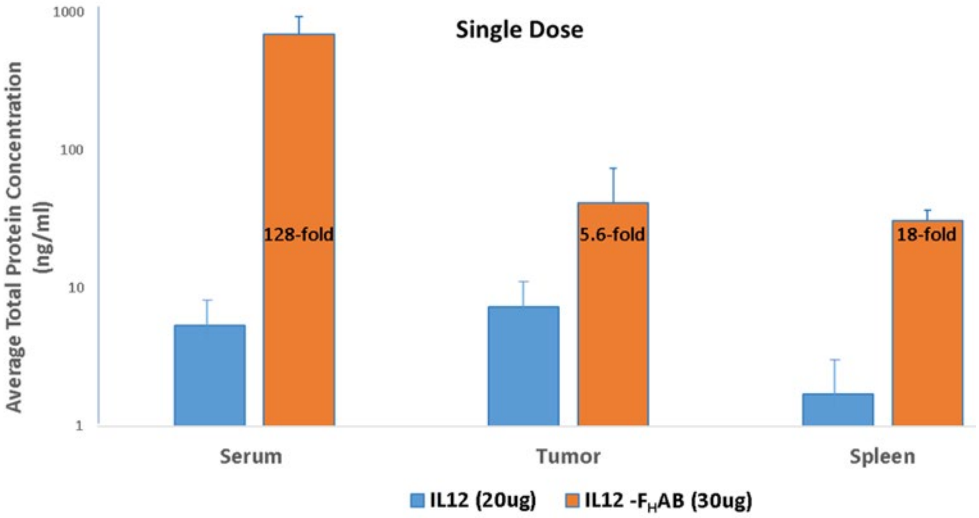

Quantitative ELISA analysis was performed on serum, tumor, and spleen tissues after a single equimolar IV dose of mIL-12 (20 ug) or mIL12-F_HAB (30 ug). The mIL-12 (blue bars) and mIL12-F_HAB (orange bars) protein concentration levels are displayed in ng/ml. Fold rise is expressed in terms of the 1.5x molar ratio.

Supplemental Figure 4: Tumor Growth for Mouse Toxicity Study of mIL-12 or mIL12-F$_H$AB

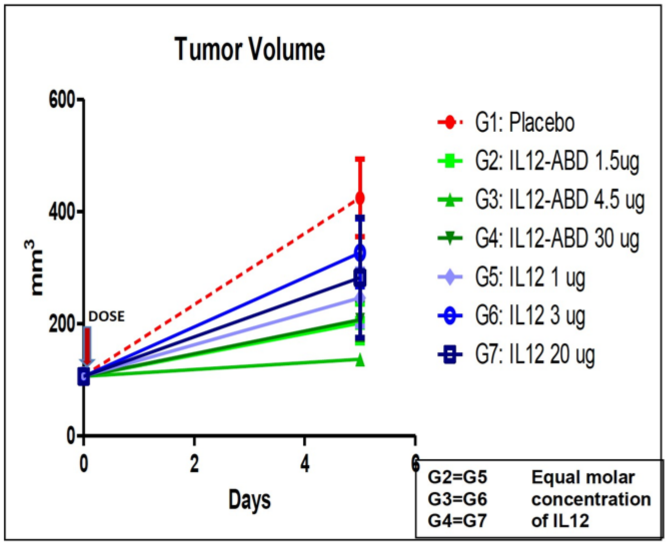

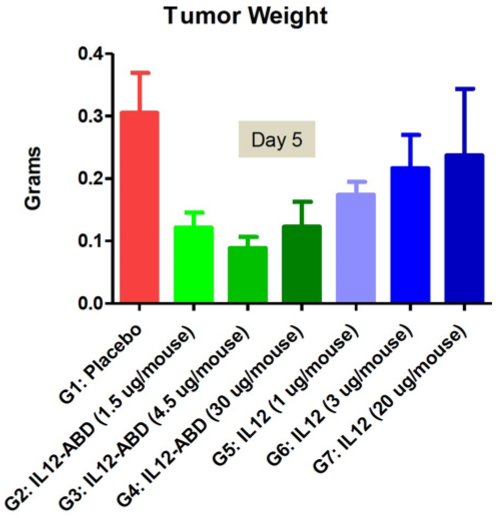

B16-F10 tumor volume on Day 5 data shows that treatment with mIL12-F$_H$AB at doses of 1.5 µg to 30 µg resulted in greater tumor reductions when compared to mIL-12.

Supplemental Figure 5: Toxicity in Mice after a Single Dose of mIL12-FHAB

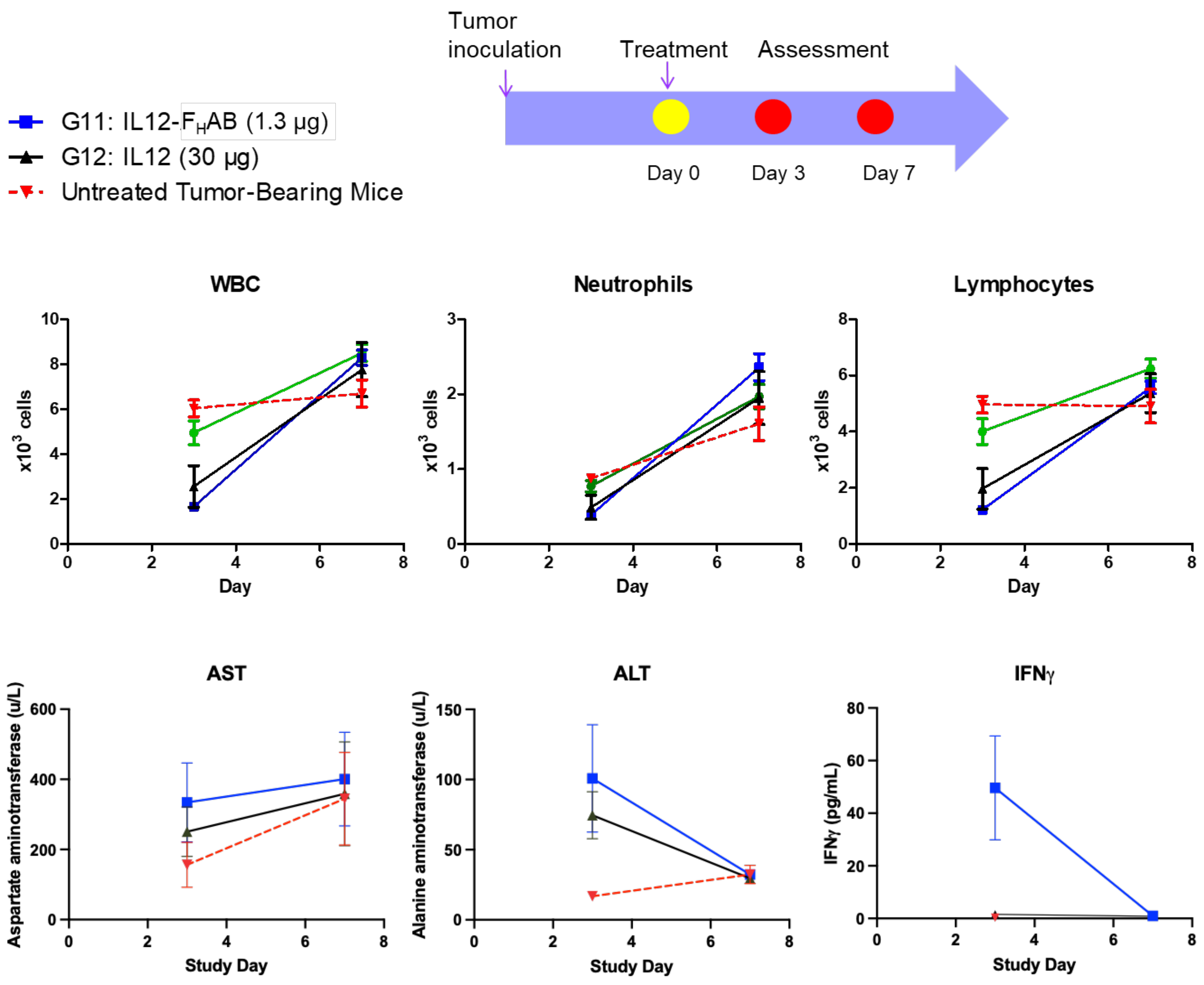

The B16-F10 mouse melanoma model was used to compare tumor growth after a low single dose of mIL12-FHAB versus a 35-fold molar equivalent dose of mIL-12. Once the tumors reached ~100-150 mm^3, groups of 10 C57BL/6 mice were administered either vehicle as a placebo, mIL-12 (30 µg as a single dose), or mIL12-FHAB (1.3 µg as a single dose) as a single IV dose on day 0. Subgroups of 4 mice were bled on Days 3 or 7 for hematology and chemistry labs.

Supplemental Figure 6: Toxicity in Mice after Three Doses of mIL12-F_HAB

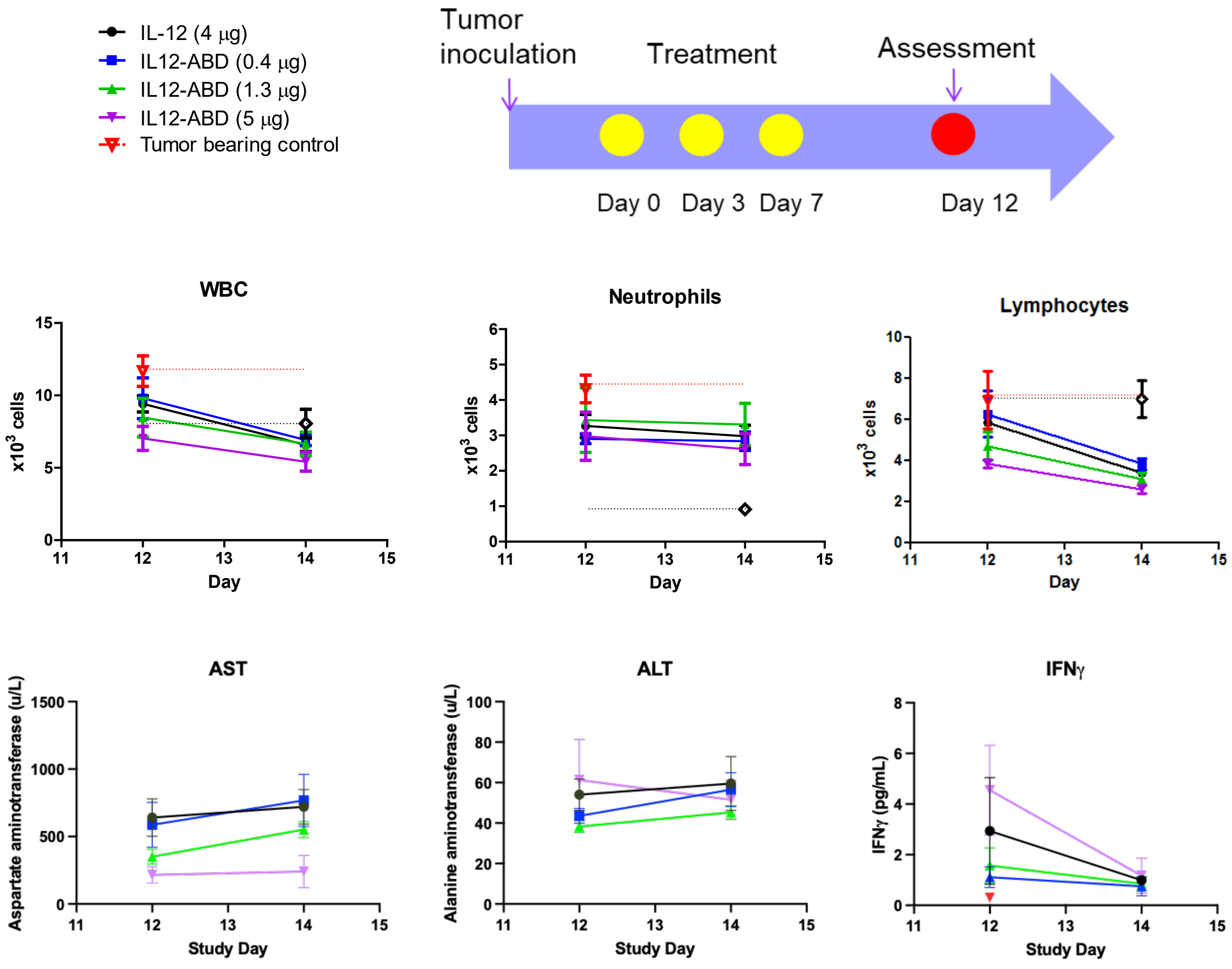

Dosing with mIL12-F_HAB three times was studied (Figure 5H), along with hematologic and liver enzyme analysis for toxicity. Once the tumors reached ~100-150 mm^3, treatments were administered as a single IV dose to 8 C57BL/6 mice per group using either vehicle as a placebo, mIL-12 (4 µg in each of 3 doses), or mIL12-F_HAB (0.4, 1.3, or 5 µg in each of 3 doses) on days 0, 3, and 7. Toxicity samples were taken on Day 12 and 14 after the first dose.

Supplemental Figure 7: GLP Toxicology in NHP: SON-1010 Concentrations in Group 4

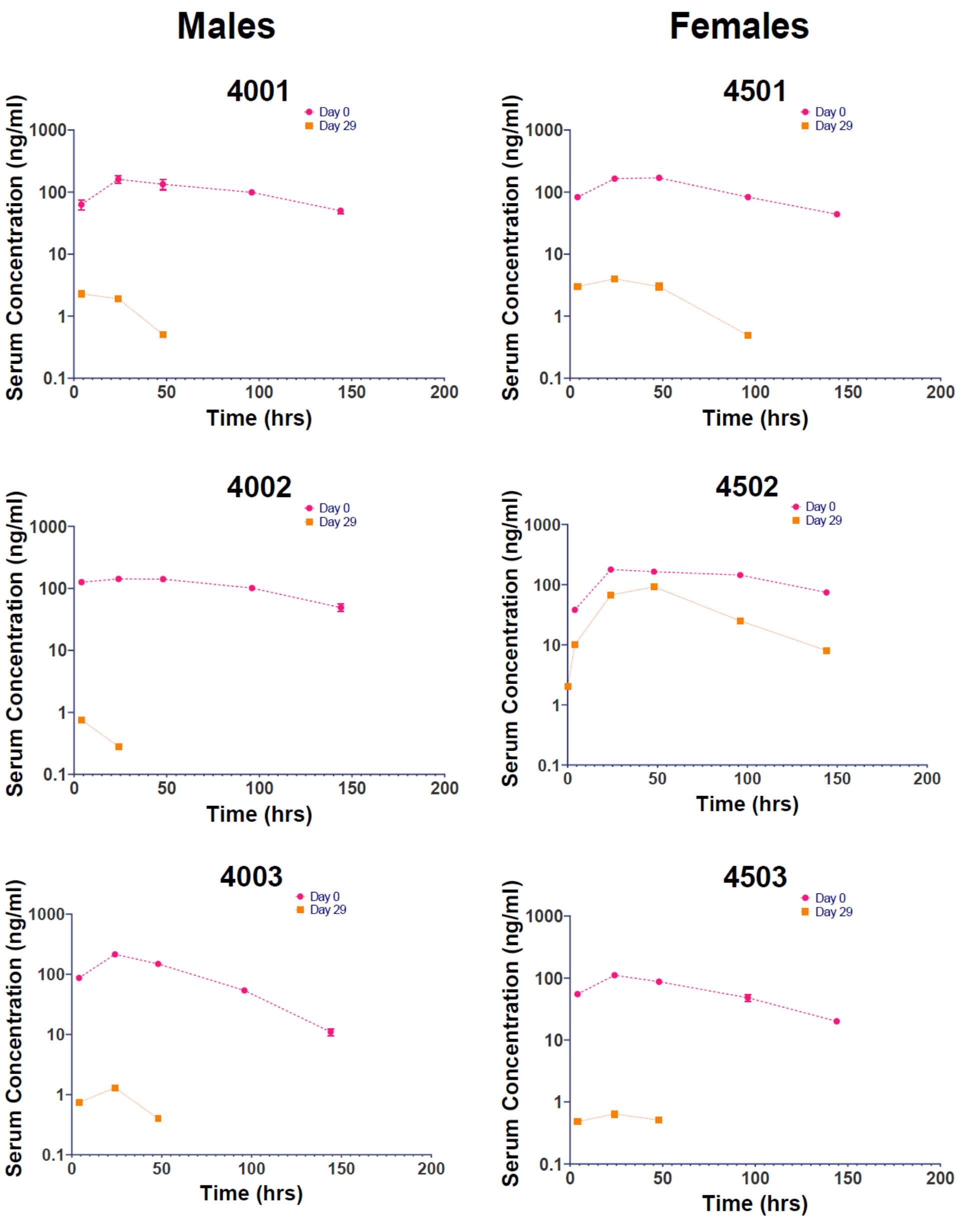

Graph of the serum concentration of SON-1010 versus time for Group 4 main study animals injected subcutaneously with SON-1010 at 0.0625 mg/kg/dose. Note that Day 1 is mislabeled as Day 0.

Supplemental Figure 8: GLP Toxicology in NHP: SON-1010 Modeling of Subcutaneous PK

NHP Mean $t_{1/2}$ (hr)	NHP Clearance (mL/hr)	NHP Volume (mL)	Clearance Human Pred. (mL/hr)	Volume Human Pred. (mL)	Human Pred. $t_{1/2}$ (hr)	Mean $t_{1/2}$ (hr)	Geometric Mean $t_{1/2}$ (hr)	Median $t_{1/2}$ (hr)	5th Percentile $t_{1/2}$ (hr)	95th Percentile $t_{1/2}$ (hr)
40.0	11.4	523.7	184.1	13,867	52.2	56.9	52.2	52.3	26.2	105.0

An exponent for CL of 0.85 for large protein molecules and an exponent of 1 for the Vd were selected, based on the molecular size of SON-1010.

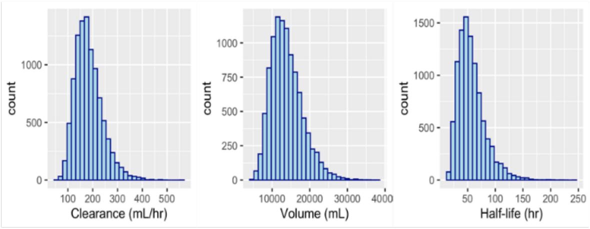

Uncertainty Approach for Calculating the Half-Life in Humans

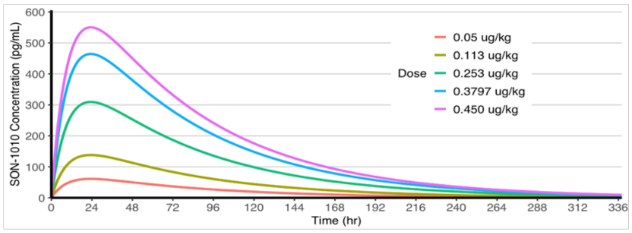

Human Single Dose SC Simulations

Allometric scaling was used with an uncertainty analysis to estimate human predicted values for clearance (CL) and the central volume of distribution (Vd) using single species scaling.

Supplemental Figure 9: GLP Toxicology in NHP: SON-1010 IgG Anti-Drug Antibodies

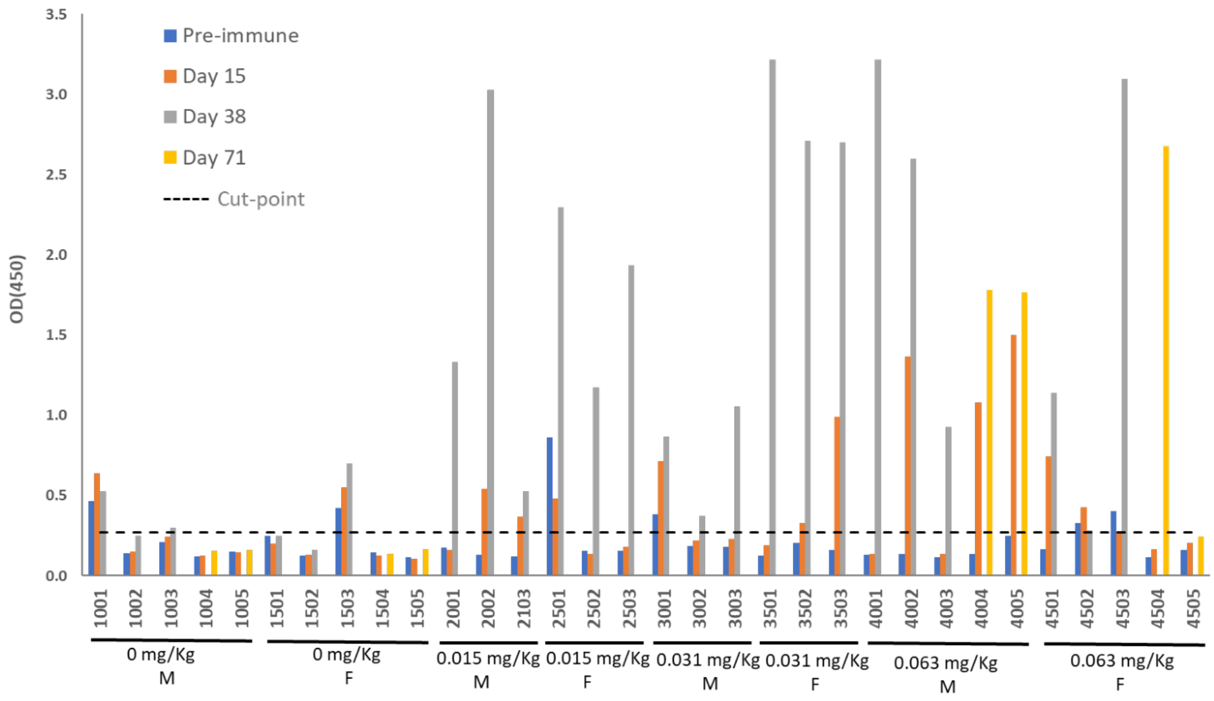

In the ADA IgG screening assay four of the 32 test monkeys showed a pre-immune IgG response slightly above the cut-point (1503, 3001, 4502 and 4503) and two monkeys had a pre-immune response significantly above the cut-point (1001 and 2501), indicating the possible presence of pre-exposure ADAs. At Day 38 (nine days after the third SON-1010 dose) or Day 71 (forty-two days after third SON-1010 dose), 20 of the 22 dosed monkeys showed IgG ADA responses significantly greater than the cut-point (all animals were not sampled at each timepoint). The cutpoint was the average of the OD450 responses from pre-immune samples + 1.645 × SD.

Supplemental Table 1: GLP Toxicology in NHP: SON-1010-related Hematology Changes

Group<br>Dose (mg/kg/dose)<br>Sex	**2**<br>**0.0156**<br>**M**	 <br>**F**	**3**<br>**0.03125**<br>**M**	 <br>**F**	**4**<br>**0.0625**<br>**M**	 <br>**F**
**RBC**						
Day 8	0.80	**0.65**	0.85	**0.76**	0.86	**0.75**
Day 15	0.87	**0.77**	0.89	**0.80**	0.80	**0.83**
Day 21	0.94	**0.83**	0.91	**0.86**	0.88	**0.87**
**HGB**						
Day 8	**0.79**	**0.65**	**0.85**	**0.74**	**0.86**	**0.77**
Day 15	**0.87**	**0.80**	0.91	**0.80**	**0.81**	**0.85**
Day 21	0.94	**0.86**	0.96	**0.88**	0.92	**0.90**
**HCT**						
Day 8	**0.77**	**0.66**	0.81	**0.71**	**0.80**	**0.75**
Day 15	**0.87**	**0.83**	0.92	**0.83**	**0.80**	**0.87**
Day 21	0.95	**0.89**	0.94	**0.89**	0.91	**0.91**
**RETIC**						
Day 8	–	–	–	–	**0.09**	–
Day 15	1.71	2.75	**2.84**	**3.43**	**3.02**	**2.78**
Day 21	1.65	2.30	2.20	2.41	**2.49**	1.91
**RDW**						
Day 15	1.13	**1.34**	1.13	**1.26**	**1.29**	1.20
Day 21	–	**1.22**	–	**1.21**	**1.26**	1.13
**PLT**						
Day 21	–	–	–	–	1.32	–
**NEUT**						
Day 8	–	–	0.29	0.49	0.47	0.51
**MONO**						
Day 8	–	**0.40**	0.34	0.58	0.49	**0.22**
**LYMPH**						
Day 8	–	–	–	**0.54**	**0.47**	**0.40**
**EOS**						
Day 8	–	–	–	**0.05**	**0.08**	0.31
**WBC**						
Day 8	–	–	**0.54**	**0.50**	**0.47**	**0.42**

M = Males; F = Females

A dash (—) indicates absence of SON-1010-related change. Numerical values indicate fold change of the treated group mean value relative to the control group mean value. Bolded values indicate the mean value was statistically different from controls at P ≤ 0.05 or P ≤ 0.01.

Supplemental Table 2: GLP Toxicology in NHP: SON-1010 related Clinical Chemistry Changes

Group<br>Dose (mg/kg/dose)<br>Sex	2<br>0.0156<br>M	 <br>F	3<br>0.03125<br>M	 <br>F	4<br>0.0625<br>M	 <br>F
**CHOL**						
Day 8	–	–	0.61	–	**0.58**	–
**TRIG**						
Day 8	3.38	2.57	1.45	2.68	2.44	2.56
**ALB**						
Day 8	**0.84**	**0.80**	**0.90**	0.81	**0.83**	0.84
Day 15	–	–	–	–	0.88	–
**GLOB**						
Day 15	–	–	–	–	1.19	–
**A/G**						
Day 8	**0.75**	**0.68**	0.83	**0.73**	0.81	**0.79**
Day 15	–	–	–	–	**0.75**	–
**CA**						
Day 8	0.94	0.93	0.95	0.93	**0.89**	0.94
Day 15	–	–	–	–	**0.92**	–
**PHOS**						
Day 8	**0.60**	–	**0.71**	–	**0.68**	**0.69**
**NA**						
Day 8	–	–	–	0.95	0.97	–
**CL**						
Day 8	–	–	–	**0.94**	**0.96**	–

M = Males; F = Females

A dash (—) indicates absence of SON-1010-related change. Numerical values indicate fold change of the treated group mean value relative to the control group mean value. Bolded values indicate the mean value was statistically different from controls at P ≤ 0.05 or P ≤ 0.01.

Supplemental Table 3: GLP Toxicology in NHP: Pharmacokinetic results for Group 4 animals*

Dose	Sex	Day	Animal ID	C_{max} (ng/ml)	C_{max}_D (kg*ng/ml/mg)	T_{max} (hrs)	T_{last} (hrs)	$T_{1/2}$ (hrs)	AUC_{last} (hrs*ng/ml)	AUC_{last}_D (hrs*kg*ng/ml/mg)	AUC_{∞} (hrs*ng/ml)	AUC_{∞}_D (hrs*kg*ng/ml/mg)	Vz_F (ml/kg)	Cl_F (ml/hrs/kg)
			4501	170	2720	48	144	49.2	15786	252576	18911	302579	234.7	3.3
			4502	178	2848	24	144	83.6	18964	303424	27891	446254	270.3	2.24
**0.0625**	**F**	**0**	4503	111	1776	24	144	45.3	9018	144288	10324	165183	395.3	6.05
			4504	176	2816	24	144	37.4	14948	239168	16512	264190	204.1	3.79
			4505	176	2816	24	144	42.0	14924	238784	16804	268858	225.5	3.72
			N	5	5	5	5	5	5	5	5	5	5	5
			Mean	162.2	2595.2	28.8	144	51.5	14728	235648	18088	289413	266	3.82
			CV%	17.7	17.7	37.3	0	35.9	24.4	24.4	35	35	28.6	36.5
			4501	4	64	24	96		244.7	3916				
			4502	92	1472	48	144	27.3	6302	100832	6617	105863		
**0.0625**	**F**	**29**	4503	1	10	24	48		26.9	431				
			4504	1	23	4	24		28.8	461				
			4505	10	160	24	144	25.2	663.1	10609	681	10900		
			N	5	5	5	5	2	5	5	2	2		
			Mean	21.6	345.92	24.8	91.2	26.2	1453.1	23250	3649	58382		
			CV%	182.8	182.8	62.9	60	5.5	187.4	187.4	115	115		
			4001	161	2576	24	144	67.5	15074	241184	19943	319089	305.2	3.13
			4002	142	2272	24	144	63.0	15736	251776	20187	322985	281.2	3.1
**0.0625**	**M**	**0**	4003	215	3440	24	144	25.5	13994	223904	14399	230387	159.9	4.34
			4004	306	4896	24	144	30.0	19356	309696	20221	323539	133.7	3.09
			4005	297	4752	24	144	26.2	17982	287712	18436	294972	128.2	3.39
			N	5	5	5	5	5	5	5	5	5	5	5
			Mean	224.2	3587.2	24	144	42.44	16428.4	262854.4	18637	298194	201.6	3.41
			CV%	33.7	33.7	0	0	49.3	13.3	13.3	13	13.3	42.1	15.7
			4001	2	37	4	48		76.5	1225				
			4002	1	12	4	24		12.5	200				
**0.0625**	**M**	**29**	4003	1	21	24	48		43.1	689				
			4004	1	16	24	48		45.4	727				
			4005	1	21	24	48		52.2	835				
			N	5	5	5	5		5	5				
			Mean	1.3	21.44	16	43.2		45.9	735				
			CV%	43.7	43.7	68.5	24.8		49.9	50				

*Data from both main study and recovery animals were grouped together for the pharmacokinetic analysis
